# A novel AI device for real-time optical characterization of colorectal polyps

**DOI:** 10.1038/s41746-022-00633-6

**Published:** 2022-06-30

**Authors:** Carlo Biffi, Pietro Salvagnini, Nhan Ngo Dinh, Cesare Hassan, Prateek Sharma, GI Genius CADx Study Group, Giulio Antonelli, Halim Awadie, Sebastian Bernhofer, Sabela Carballal, Mário Dinis-Ribeiro, Agnès Fernández-Clotet, Glòria Fernández Esparrach, Ian Gralnek, Yuta Higasa, Taku Hirabayashi, Tatsuki Hirai, Mineo Iwatate, Miki Kawano, Markus Mader, Andreas Maieron, Sebastian Mattes, Tastuya Nakai, Ingrid Ordas, Raquel Ortigão, Oswaldo Ortiz Zúñiga, Maria Pellisé, Cláudia Pinto, Florian Riedl, Ariadna Sánchez, Emanuel Steiner, Yukari Tanaka, Andrea Cherubini

**Affiliations:** 1 Artificial Intelligence Group, Cosmo AI/Linkverse, Lainate/Rome, Italy; 2 Department of Biomedical Sciences, Humanitas University, Via Rita Levi Montalcini 4, 20072 Pieve Emanuele, Milan Italy; 3 Endoscopy Unit, Humanitas Clinical and Research Center IRCCS, Rozzano, Italy; 4 VA Medical Center, Kansas City, MO USA; 5 University of Kansas School of Medicine, Kansas City, MO USA; 6 Milan Center for Neuroscience, University of Milano–Bicocca, 20126 Milano, Italy; 7 Gastroenterology and Digestive Endoscopy Unit, Ospedale dei Castelli (N.O.C.), Ariccia, Italy; 8 Gastrointestinal and Liver Institute, Emek Medical Center, Afula, Israel; 9 Gastroenterology and Hepatology and Rheumatology, University Hospital of St. Pölten, St. Pölten, Austria; 10 Gastroenterology Department, Hospital Clinic of Barcelona, Barcelona, Spain; 11 Gastroenterology Department, Portuguese Oncology Institute of Porto, Porto, Portugal; 12 Department of Gastroenterology, Kita-Harima Medical Center, Ono City, Japan; 13 Gastroenterology Department, Hyogo Cancer Center, Hyogo, Japan; 14 Gastroenterology Department, Sugita Genpaku Memorial Obama Municipal Hospital, Obama, Japan; 15 Gastrointestinal Center, Sano Hospital, Hyogo, Japan; 16 Kobe Red Cross Hospital, Hyogo, Japan; 17 Kobe University Hospital, Hyogo, Japan

**Keywords:** Colonoscopy, Image processing

## Abstract

Accurate in-vivo optical characterization of colorectal polyps is key to select the optimal treatment regimen during colonoscopy. However, reported accuracies vary widely among endoscopists. We developed a novel intelligent medical device able to seamlessly operate in real-time using conventional white light (WL) endoscopy video stream without virtual chromoendoscopy (blue light, BL). In this work, we evaluated the standalone performance of this computer-aided diagnosis device (CADx) on a prospectively acquired dataset of unaltered colonoscopy videos. An international group of endoscopists performed optical characterization of each polyp acquired in a prospective study, blinded to both histology and CADx result, by means of an online platform enabling careful video assessment. Colorectal polyps were categorized by reviewers, subdivided into 10 experts and 11 non-experts endoscopists, and by the CADx as either “adenoma” or “non-adenoma”. A total of 513 polyps from 165 patients were assessed. CADx accuracy in WL was found comparable to the accuracy of expert endoscopists (CADx_WL_/Exp; OR 1.211 [0.766–1.915]) using histopathology as the reference standard. Moreover, CADx accuracy in WL was found superior to the accuracy of non-expert endoscopists (CADx_WL_/NonExp; OR 1.875 [1.191–2.953]), and CADx accuracy in BL was found comparable to it (CADx_BL_/CADx_WL_; OR 0.886 [0.612–1.282]). The proposed intelligent device shows the potential to support non-expert endoscopists in systematically reaching the performances of expert endoscopists in optical characterization.

## Introduction

Colorectal cancer is one of the most common malignancies^1^. Optical colonoscopy with white light (WL) endoscopy is the gold standard for the detection and resection of colorectal mucosal polyps and its adoption in population-based screening programs has resulted in a significant reduction in the incidence and mortality of colorectal cancer^2^. Accurate real-time visual differentiation between adenomatous and non-adenomatous polyps (optical characterization, OC) during colonoscopy is clinically relevant to select optimal treatment regimen, avoid inappropriate endoscopic resection, improve cost-effectiveness, and reduce the number of polypectomies^3,4^.

In order to standardize OC, several classification schemes have been proposed with the aim of being incorporated into clinical practice^5–7^. These classifications are based on combinations of vascular and mucosal patterns, specific features of the polyp surface, and the presence of a cloudy or irregular appearance and indistinct borders. Moreover, although optical colonoscopy is performed using WL large spectrum illumination, all these classification schemes are based on virtual chromoendoscopy illumination (narrow-spectrum blue light [BL]^8^) able to enhance the appearance of superficial mucosal vascular patterns. Nevertheless, these classifications in BL showed significant inter- and intra-observer variability when prospectively evaluated, limiting their widespread adoption by the endoscopic community^9,10^.

Endoscopy procedures are an ideal arena for the development of intelligent medical devices^11,12^. This is due to the huge quantity of information that the physician needs to extract and interpret from the video flow, in real-time, under time pressure, and with repetitive modalities during long working hours. In similar situations, where humans may act non-Bayesian by violating probabilistic rules and thus making inconsistent decisions, artificial intelligence (AI) has proven to be a valuable tool to help humans in making better decisions^13^. The first generation of AI-based medical devices in colonoscopy authorized by regulatory bodies has focused on improving the task of polyp detection^14,15^. Different randomized controlled trials have demonstrated the ability of such computer-aided detection (CADe) devices to improve the detection of precancerous polyps during colonoscopy^16–19^. However, AI-based algorithms in endoscopy have also the potential of supporting physicians in the task of OC (CADx), thereby reducing the limitations described above.

Nevertheless, AI algorithm proposed for the task of OC has failed to be implemented in mainstream clinical practice so far^20–22^. This might be due to limitations in design that prevent seamless integration into clinical workflow, such as classifying still images rather than videos^22^, or requiring additional technology such as virtual chromoendoscopy (BL) or endocytoscopy as a prerequisite to operate^21^.

In this work, we propose an intelligent medical device for real-time OC of colorectal polyps that can overcome the limitations of current solutions and can be integrated easily into clinical workflow. The device can operate on an unaltered conventional WL video stream without human intervention. We validate this AI on a prospectively acquired dataset with a multi-reader study design. For this purpose, we benchmark the performance of the AI against a group of expert endoscopists and a group of non-expert endoscopists. Our hypotheses are that AI accuracy is comparable to experts and superior to non-experts, with a substantial equivalence between performances in WL illumination and virtual chromoendoscopy (BL). Our predictions were pre-registered before the start of the data gathering, together with the study plan and statistical models and analyses (available at https://osf.io/m5cxt).

## Results

**Fig. 1:**
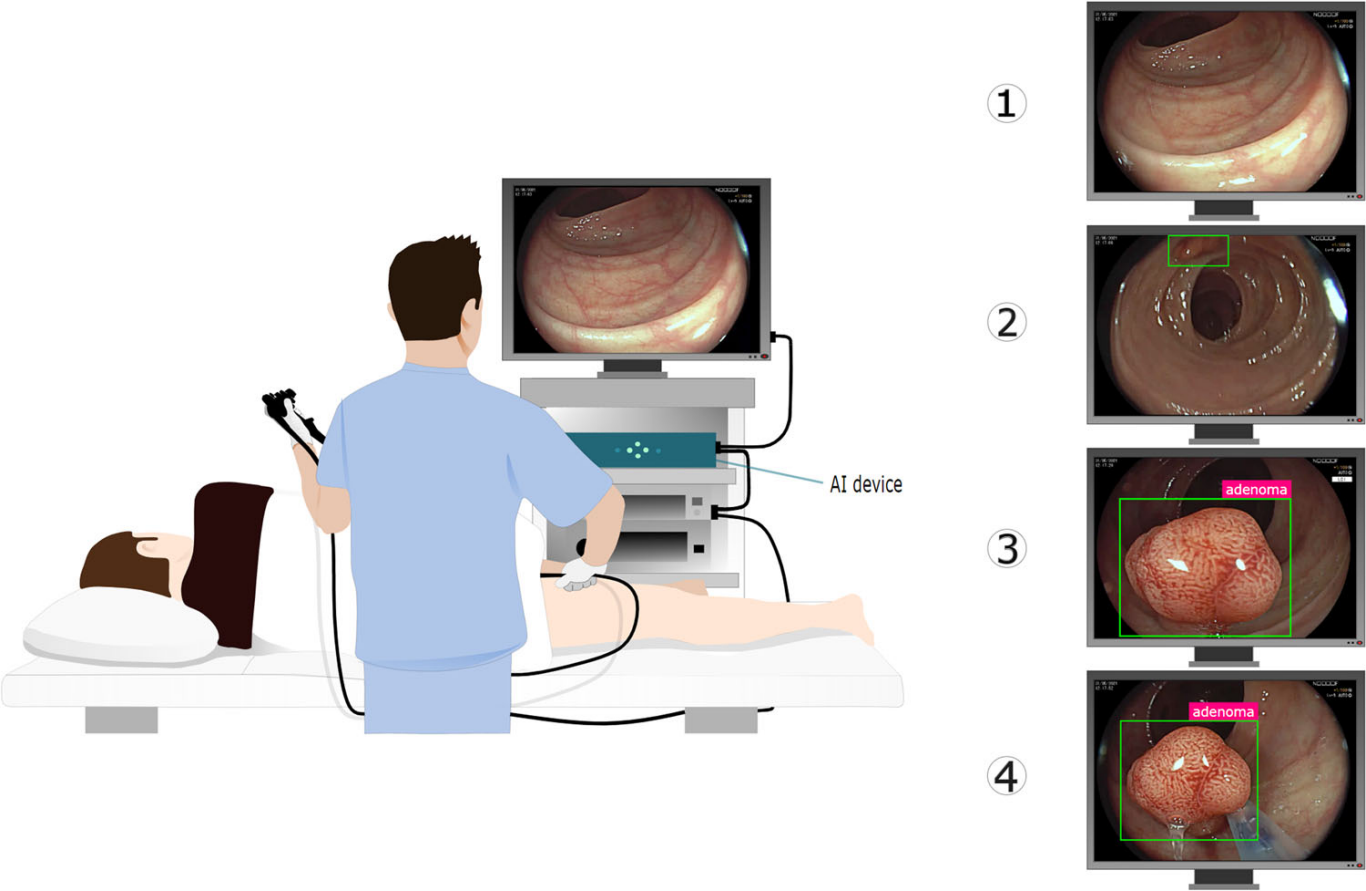
AI device intended use workflow. The endoscopy video stream flows through the device with no modification or delay (<1.5 μs). In real time (50–60 ms), the device augments the video stream by adding overlay markers to surround areas of interest, such that they can be further inspected by the endoscopist. During colon navigation (1), the endoscopist is focused on exposing the mucosa appropriately in order to facilitate the task of polyp detection. The AI intelligent device activates when a polyp is detected (2, CADe). At this stage, the endoscopist examines the mucosa in order to characterize the lesion and decide on clinical action: if a polyp is framed consistently by the endoscopist, the CADx activates automatically (3) and the histology prediction is added to the green box. After possible endoscopic resection of the polyp (4), navigation is resumed (1) and CADx automatically disengages. These tasks are iterated as a loop until the end of the procedure.

Figure 1 depicts the intended use in the clinical workflow of the proposed intelligent device. Briefly, the CADx system is designed to automatically activate when a new polyp is detected by a CADe detection algorithm in a colonoscopy video stream. For each polyp, the device overlays a frame-by-frame live decision specifying its binary histology (“adenoma” or “non-adenoma”). The CADx can also abstain from predicting the polyp histology in a frame either by printing “no-prediction” if the system is unsure about the histology or “analyzing” if an insufficient number of features across multiple frames was detected. Example videoclips of the CADx real-time output for three polyps of the study are provided as Supplementary Videos.

This study included lossless video recording and histology information on 513 prospectively acquired colorectal polyps (198 adenomas, 315 non-adenomas) in a total of 165 subjects (77 males, 88 females, mean age 66.6 ± 10.2). The proposed medical device was applied to each full-procedure video recording, hence reproducing the same frame-by-frame output that was shown in the clinical room. The full per frame processing time of the device (including on-screen output visualization) was always inferior to 60 ms, while the average CADx processing time was 2 ms—as per the device specifications. A total of 10 experts and 11 non-experts endoscopists performed OC of each polyp via an online platform enabling careful assessment of the video recordings. Video recordings contained imaging of the polyp using both WL and BL technology. The expert reviewer group had an experience (measured in years of activity) of 12.3 ± 7.3 years [range: 6–29 years], while the non-expert reviewer group had an experience (measured in number of colonoscopies performed) of 363 ± 136 colonoscopies [range: 100–500 colonoscopies, range of years of experience: 1–3 years].

### Performance comparisons

Study endpoints were evaluated using both log-binomial regression and bootstrapping methods. Table 1 shows the results of group comparisons using log-binomial regression. In detail, CADx accuracy in WL was found to be non-inferior to the accuracy of expert endoscopists (CADx_WL_/Experts; OR 1.211 [0.766–1.915]; *p* < 0.001) using histopathology as a reference standard. Moreover, CADx accuracy in WL was found superior to the accuracy of non-expert endoscopists (CADx_WL_/Non-experts; OR 1.875 [1.191–2.953]; *p* = 0.003), and CADx accuracy in BL was found non-inferior to it (CADx_BL_/CADx_WL_; OR 0.886 [0.612–1.282]; *p* = 0.003). Performances of individual reviewers are reported in Supplementary Results 4.

**Table 1:** Experts, non-experts, and CADx performance metrics for optical characterization.

	Accuracy	Sensitivity	Specificity	Undetermined
Group experts	82.2% [80.1–84.2]	76.6% [72.8–80.1]	86.6% [82.7–89.7]	1.4% [0.6–2.5]
Group non-experts	74.9% [72.4–77.3]	72.4% [59.4–82.4]	80.9% [73.0–86.9]	1.9% [0.8–3.1]
CADx white light	84.8% [81.3–87.8]	80.7% [74.2–85.7]	87.3% [83.0–90.6]	6.2% [4.3–8.4]
CADx blue light	83.2% [79.0–86.7]	81.7% [74.5–87.2]	84.1% [78.8–88.3]	28.1% [24.2–31.8]

CADx output is provided separately for video frames in white light and video frames in blue light. Expert (10 reviewers) and non-expert (11 reviewers) assessed both white light and blue light video frames. Accuracy, sensitivity, and specificity are calculated on the fraction of 513 polyps for which a prediction was provided, using histopathology as the reference standard. The column labeled “Undetermined" displays the percentage of polyps for which the reviewers or CADx failed to provide a prediction, respectively.

**Fig. 2:**
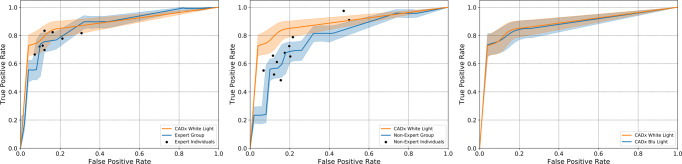
ROC curves calculated using the bootstrap method. From left to right: CADx white light vs non-expert endoscopists, CADx white light vs expert endoscopists, and CADx white light vs CADx blue light. The light band around the ROC curves represents 95% confidence intervals derived by bootstrapping. Black dots represent individual reviewers' performances.

Figure 2 shows the results of group comparisons using the bootstrap method. In detail, the area under the curve (AUC) for CADx in WL (AUC_WL_: 0.8653 [0.8304–0.8967]) was found non-inferior to that of expert endoscopists (AUC_Exp_: 0.8553 [0.8203–0.8881]). Moreover, CADx accuracy in WL was found superior to the accuracy of non-expert endoscopists (AUC_NonExp_: 0.7769 [0.7356–0.8171]), and CADx accuracy in BL was found non-inferior to it (AUC_BL_: 0.8545 [0.8141–0.8915]).

### Reviewer/CADx/histology agreement

**Fig. 3:**
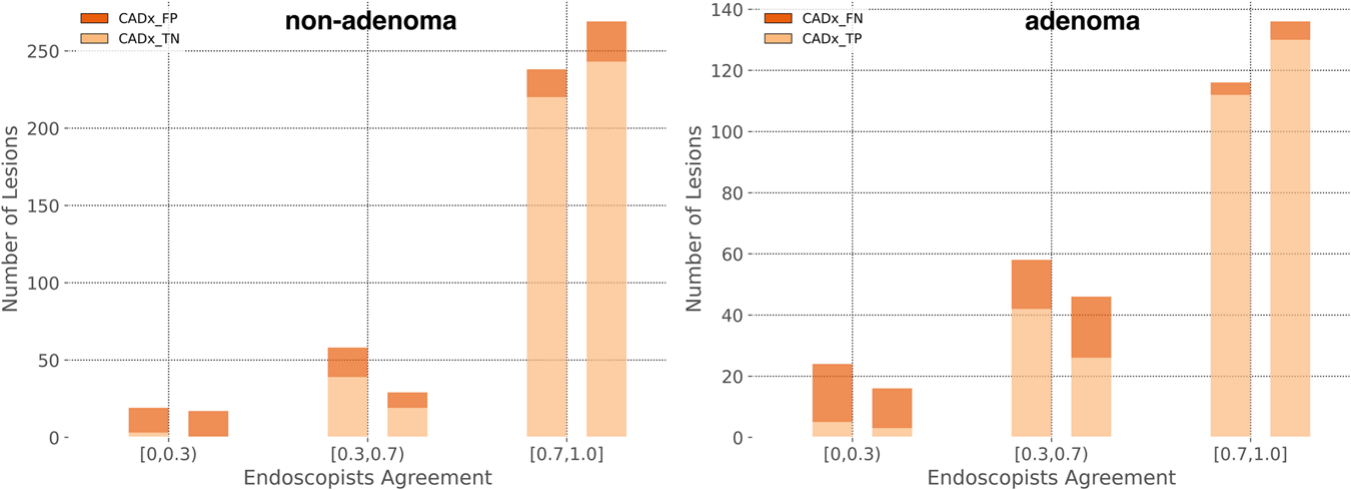
Graphical visualization of agreement between reviewer predictions and histology. Results for polyps with non-adenomatous and adenomatous histology are reported in left and right plots, respectively. In each box, the rightmost pair of bins reports polyps for which 70–100% of the endoscopists' predictions are in agreement with histology, while the leftmost pair of bins reports polyps for which less than 30% of the endoscopists' predictions are in agreement with histology. Each bin pair reports non-experts on the left and experts on the right. The distribution suggests that most of CADx FPs and FNs are polyps where reviewers disagree with each other, or are unanimously in disagreement with histology.

**Fig. 4:**
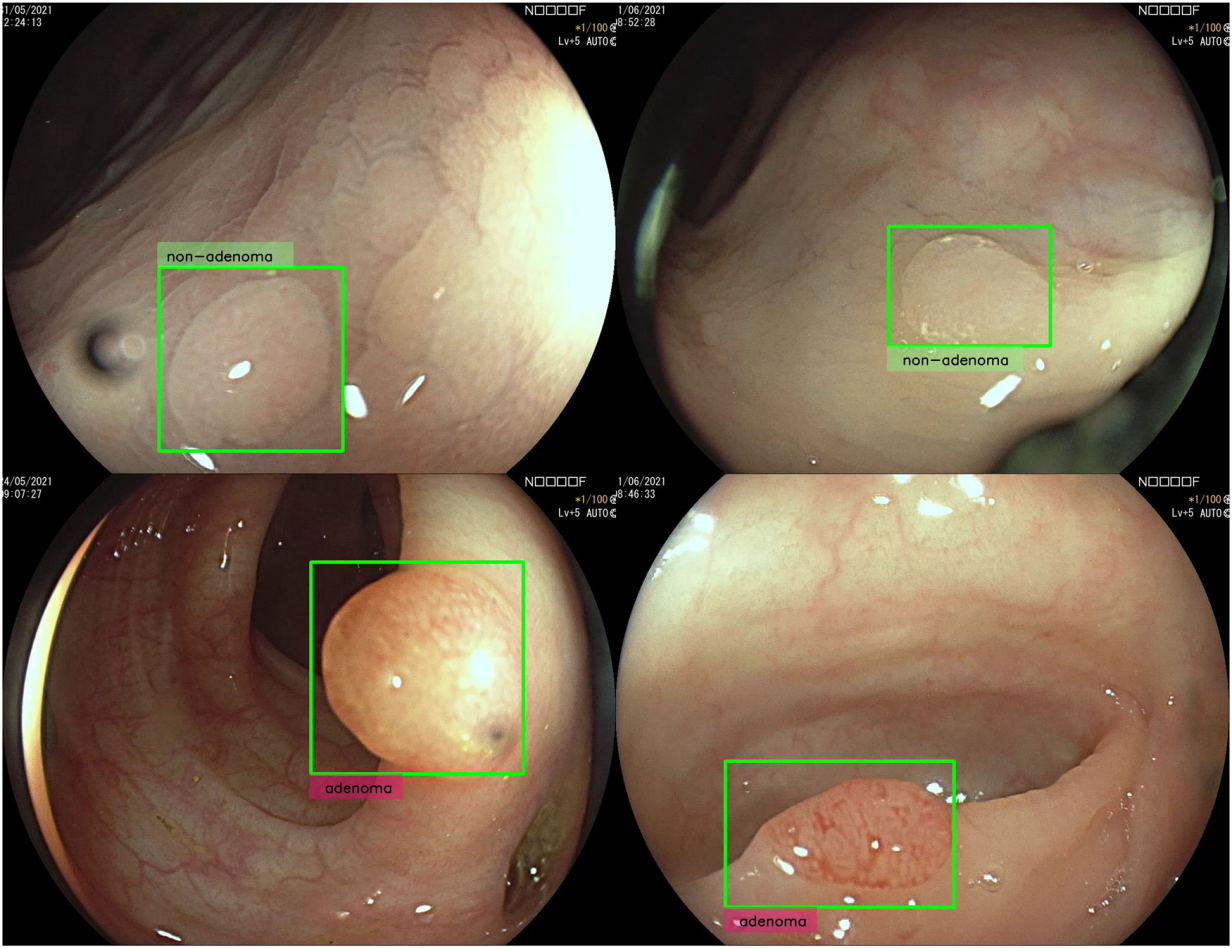
Exemplar images of polyps with disputed histology. Video frames of polyps for which both CADx and all reviewer predictions are in contrast with the histology ground truth. These polyps are considered false negatives (top row—histology: adenoma, CADx prediction: non-adenoma) or false positives (bottom row—histology: non-adenoma, CADx prediction: adenoma) in the reported accuracies.

Figure 3 shows the agreement of expert and non-expert endoscopists with ground truth, computed as the fraction of endoscopists correctly predicting the polyp histology. Notably, both experts and non-expert endoscopists were unanimously in disagreement with the ground truth for nine non-adenomatous polyps (five diminutive (≤5 mm), two small (6–9 mm), and two large (≥10 mm)) and six adenomatous (all diminutive) polyps. CADx classified the same nine non-adenomas and six adenomas in disagreement with histology and in agreement with endoscopists. Figure 4 shows example images of such polyps.

## Discussion

Compared to previous studies in this area, our study contributed uniquely in the following aspects: we have developed for the first time an intelligent medical device that can perform the task of OC in real-time on unaltered conventional WL videos; the device does not need additional technology such as virtual chromoendoscopy on the endoscopy tower, that might slow down the clinical workflow; we have validated the device using a prospectively acquired dataset with a multi-reader design.

In clinical practice, the task of OC performed during live colonoscopy is not a static assessment of a polyp portrait, but rather a fluid and dynamic process of decision build-up in the endoscopist’s brain. This process is heavily affected by polyp appearance and its morphological characteristics (i.e., location relative to folds, level of cleansing, size, etc.). Thus, the time needed to complete the assessment of a single polyp can vary wildly, ranging from a fraction of a second to several minutes. During this examination, it is very frequent for the endoscopist to change opinion for a given polyp, possibly jumping from adenoma to non-adenoma or vice-versa, whenever a particular illumination or viewing angle highlights a feature of a specific class. Consequently, the process of OC can be considered as a weighted average of different features over time.

Previous studies on OC CADx failed to capture this dynamic process and likely over-inflated reported performances for several reasons. First, previous trials focused on the classification of single images, with the physician asked to freeze the video during live endoscopy, and subsequently submit the selected frame to the image classifier^21,22^. This approach introduces a bias since the physician, especially if non-expert, might not select the most representative image of a polyp. Moreover, selecting a single image would fail to represent the complex variability of observing a polyp from different viewpoints, zoom, or illumination angle. For the very same reasons, assessing the performances of CADx using the classification of human-chosen snapshot or short videoclips of polyps^20^ is likely to over-inflate performances, since the CADx would be trained and validated only on near-perfect images. Second, previous studies required additional technologies such as endocytoscopy^21^, or proprietary virtual chromoendoscopy illuminations, preventing generalizability of results. Moreover, these approaches require the physician to manually engage and disengage the OC module, thus requiring additional steps during the normal succession of tasks in real-life colonoscopy.

In order to be used in a real-world setting, an AI device should integrate seamlessly into clinical practice. For this purpose, the intelligent device in this work is designed to engage automatically when a polyp is framed consistently, thus “interpreting” the wish of the physician to know more about the imaged region (Fig. 1). By using the very same mechanism, it disengages automatically when normal navigation is resumed. This feature is possible because in our device the image classification is a process of the cascade to the detection process, thus it can “follow” the polyp as it moves around the image frame. Other CADx algorithms that are not linked to a detection module (CADe) are bound to output a classification on the entire image. An additional benefit of this design is the ability to track and characterize different polyps even when these appear simultaneously on the image frame.

Another important finding of our study was the ability to reach very high accuracies using conventional wide spectrum WL illumination, indirectly showing that WL delivers all information needed to be as accurate as experts who use BL technology.

A noteworthy result of this study was the disagreement observed between a large panel of endoscopists with heterogeneous expertise and histology (Fig. 3). A similar phenomenon, although measured considering the output of a single very expert senior endoscopist, has been reported recently^23,24^. The reasons for this disagreement could be multiple and related to specimen retrieval and subsequent processing, rather than a misdiagnosis. This could explain our observation that this effect is exacerbated for diminutive polyps (≤5 mm) for which manual handling is more difficult and thus more prone to error. The observation led Shahidi et al.^24^ to question pathology as the gold standard for assessing diminutive colorectal polyps. Although this position could be questioned^25^, it suggests for AI a potential arbitration role when endoscopist and pathologist assessments of the same polyp diverge.

This work has limitations. First, the dataset acquired for performance assessment originates from a single center, although using different endoscope manufacturers. Second, the current version of CADx characterizes polyps according to a two-class model that includes sessile serrated polyps into the non-adenoma class^26^, forcing the endoscopist to look for serrated features likewise happens in WASP criteria^27^. Although identifying sessile serrated polyps as a separate class could be beneficial, the current size of the dataset used for training the CADx does not support a three-class model with reliable accuracies. Future CADx releases including this category as a separate output are warranted.

Real-time colonoscopy is a fertile area for developing intelligent devices that are able to effectively allocate tasks between humans and AI, thereby achieving a superior outcome by aggregating the output of its parts. In this context, the medical device described in this study may allow non-experts to leverage the predictive power of AI while using their own knowledge to make a choice from the predictions of the AI. In conclusion, this device offers the potential to standardize the practice of OC and to ensure in all colonoscopies the same accuracy that can be met only by a few very experienced expert physicians.

## Methods

### CADx system

The CADx system comprises two online algorithms working on the outputs of two convolutional neural network models. The first convolutional neural network model is named Polyp Characterization Network and it has a two-fold purpose: (1) to classify each detected polyp in a single video frame as “adenoma” or “non-adenoma” polyp and (2) to provide a polyp image appearance descriptor for each detected polyp in the current frame to be used for polyp tracking. The second convolutional neural network, named Polyp Imaging Quality Network, is responsible for providing an imaging quality score to each detected polyp expressing how clearly the polyp features are imaged in the current video frame. This second network is needed since low-quality images can introduce noise in the spatial-temporal reasoning of the CADx. The first online algorithm is responsible for polyp tracking across multiple frames, while the second is an online temporal aggregation algorithm that aggregates frame-by-frame classification and imaging quality information for each tracked polyp and provides a live decision based on a moving temporal window.

### Polyp characterization network

Given an input frame ${{{{\mathcal{I}}}}}_{t}$ at time *t* of a regular colonoscopy video and a set of *N* polyp detections ${{{{\mathcal{B}}}}}_{t}={\left\{{{{{\bf{b}}}}}_{t,i}\right\}}_{i = 1}^{N}$ as detected by any polyp detection model on that frame, the first component of the proposed AI system is a learning-based model that learns from data the mapping between the image content of each bounding box **b**_*t*,*i*_ to the histology *h*_*i*_ of the polyp it contains. We employ ResNet18, a deep convolutional neural network commonly used for classifying histopathological images^28^, for this task. In order to input the polyp images at an appropriate resolution while providing some contextual information, the input of the characterization network is an image ${{{{\mathcal{X}}}}}_{t,i}$ resulting from the cropping of the input frame ${{{{\mathcal{I}}}}}_{t}$ around the bounding box **b**_*t*,*i*_ plus a 50 pixels margin, which is rescaled to 512 × 512 size. The output of the characterization network is a score *c*_*t*,*i*_, between [0,1], expressing the probability of the content of the bounding box to be an adenoma (1) or a non-adenoma (0) polyp. By applying the classification network to all the N detections in a frame, a set of characterization scores ${{{{\mathcal{C}}}}}_{t}={\left\{{c}_{t,i}\right\}}_{i = 1}^{N}$ can hence be obtained. The characterization model is trained using binary classification cross entropy (CE) as a loss function and the ground-truth histology is represented as a two-dimensional vector **y**_*t*,*i*_ while its predicted value as softmax scores ${\hat{{{{\bf{y}}}}}}_{t,i}$, ${{{{\mathcal{T}}}}}_{cl}={{{{\mathcal{T}}}}}_{CE}({\hat{{{{\bf{y}}}}}}_{t,i},{{{{\bf{y}}}}}_{t,i})$. Mixup training method was adopted to provide a better-calibrated network and to reduce overfitting^29^.

### Polyp re-identification algorithm

The task of OC is performed by a human by considering many subsequent frames before expressing a decision. The proposed AI system aims at mimicking this decision-making process by producing a frame-by-frame temporally weighted decision for each detected polyp when enough confidence about a prediction has been acquired. In order to achieve this, an important milestone is to be able to follow a polyp across multiple frames in a colonoscopy video.

In our system, we propose an online polyp re-identification algorithm that both exploits single-frame polyp appearance and spatio-temporal information for this task. In order to extract single-frame polyp appearance information, we modify the characterization network so that an 8k-dimensional feature descriptor **f**_*t*,*i*_ for each input cropped image ${{{{\mathcal{X}}}}}_{t,i}$ can be extracted. Specifically, we make use of a multi-task learning approach by attaching at the end of the characterization network encoder a second convolutional neural network branch. In this way, the network learns how to encode and reconstruct each input ${{{{\mathcal{X}}}}}_{t,i}$ by only using its 8k-dimensional descriptor **f**_*t*,*i*_ and for each frame at set of bounding box image appearance descriptors ${{{{\mathcal{F}}}}}_{t}={\left\{{{{{\bf{f}}}}}_{t,i}\right\}}_{i = 1}^{N}$ can be obtained. This second network branch is trained end-to-end with the network classification network by means of a reconstruction loss ${{{{\mathcal{L}}}}}_{rec}={{{{\mathcal{L}}}}}_{MSE}({\hat{{{{\mathcal{X}}}}}}_{t,i},{{{{\mathcal{X}}}}}_{t,i})$, which is pixel-wise mean squared error in the RGB space between the input image *X*_*t*,*i*_ and its reconstruction ${\hat{X}}_{t,i}$. As a consequence, the overall loss for the classification network becomes ${{{{\mathcal{L}}}}}_{tot}={{{{\mathcal{L}}}}}_{rec}+{{{{\mathcal{L}}}}}_{cl}$.

The proposed re-identification algorithm outputs at each time *t* a set ${{{{\mathcal{T}}}}}_{t}={\{{{{{\bf{L}}}}}_{j}\}}_{j = 1}^{{N}_{at}}$ of *N*_*at*_ actively followed polyps. Each element **L**_*j*_ is a set representing a polyp history by means of its bounding box coordinates ${\left\{{{{{\mathcal{B}}}}}_{k}\right\}}_{k = 1}^{K}$ and the corresponding appearance vectors ${\left\{{{{{\mathcal{F}}}}}_{k}\right\}}_{k = 1}^{K}$ and classification scores ${\left\{{{{{\mathcal{C}}}}}_{k}\right\}}_{k = 1}^{K}$ across all the time frames *k* in which the polyp was found since it is actively followed. The set of actively followed polyps ${{{{\mathcal{T}}}}}_{t}$ is obtained by assigning the polyps detected at frame *t*, ${{{{\mathcal{B}}}}}_{t}$, to the set of actively followed polyps ${{{{\mathcal{T}}}}}_{t-1}$ of the previous frame by exploiting the Hungarian (Kuhn–Munkres) algorithm^30^. In particular, the proposed algorithm first tries to associate each polyp detection in ${{{{\mathcal{B}}}}}_{t}$ to each actively followed polyp by means of a spatial assignment, then, in a second step, by means of an appearance-based assignment. Both assignments are obtained by solving an unbalanced linear assignment problem given the corresponding cost matrices. The cost matrix of the spatial-based assignment is computed by the IoU between each new detection and the last detected bounding box for each active polyp, while the cost matrix of the appearance-based assignment is computed using the cosine distance between the appearance features ${{{{\mathcal{F}}}}}_{t}$.

### Online temporal aggregation algorithm

The online temporal aggregation algorithm is responsible for printing live, on each frame *t*, a characterization decision for each visible box ${{{{\mathcal{B}}}}}_{t}$ in the list of the followed polyps ${{{{\mathcal{T}}}}}_{t}$. The algorithm is applied after having computed ternary quality scores **q**_*i*,*t*_ for the N detections in the current frame via the Polyp Imaging Quality Network. Four types of different predictions can be produced by the algorithm: “adenoma", “non-adenoma", “no-prediction" or “analysing". “analysing" is printed near the polyp to communicate to the endoscopist to keep imaging the target polyp until the minimum number *N*_*m*_ of frames is reached. The value of *N*_*m*_ was chosen so that the algorithm could take into account a sufficient number of frames to produce an OC prediction while at the same time causing only a short, affordable delay in the prediction from when the polyp was first detected. When the minimum number of frames *N*_*m*_ is reached, the number of non-adenoma and adenoma frame by frame predictions are computed with the introduction of two hyperparameters *δ*_low_ and *δ*_high_ that define when a frame by frame prediction has low confidence: *N*_*na*_ = ({*c*_*k*,*j*_ ∈ {**L**_*j*_}∣(*c*_*j*_ < 0.5 − *δ*_low_) ∧ **q**_*i*,*t*_ ≥ 1} and *N*_*a*_ = {*c*_*k*,*j*_ ∈ {**L**_*j*_}∣(*c*_*j*_ > 0.5 + *δ*_high_) ∧ **q**_*i*,*t*_ ≥ 1}. If *N*_*na*_ or *N*_*a*_ is the majority of the total number of valid frames the algorithm prints “adenoma" or “non-adenoma" on the bounding box, otherwise “no-prediction" is printed.

### CADx training

The Polyp Characterization Network and the Polyp Imaging Quality Network were trained using data extracted from the study “The Safety and Efficacy of Methylene Blue MMX Modified Release Tablets Administered to Subjects Undergoing Screening or Surveillance Colonoscopy” (ClinicalTrials.gov NCT01694966) a multinational, multicenter study that enrolled over 1000 patients. The study recorded lossless, high-definition, full-procedure colonoscopy videos and complete information on polyp characteristics and histology. The histopathological evaluation was based on the revised Vienna classification of gastrointestinal epithelial neoplasia^26^. Polyps corresponding to Vienna category 1 (negative for neoplasia) or 2 (indefinite for neoplasia) were considered “non-adenoma”. Polyps corresponding to Vienna category 3 (mucosal low-grade neoplasia), 4 (mucosal high-grade neoplasia), or 5 (submucosal invasion of neoplasia), were considered “adenoma”. To avoid any possible histology mismatch in the case multiple polyps appeared and were biopsied in the field-of-view at the same time, we chose to exclude from the dataset all polyps appearing simultaneously or in close succession. The video dataset thus obtained was further split into training, validation, and test subsets and frames containing a polyp were manually annotated by trained personnel with patients/polyps/images distributed as follows: 345/957/63,445 (training), 44/133/8645 (validation), and 165/405/26,412 (test).

### CADx device

The proposed CADx feature was implemented for GI Genius v2.0 (developed by Cosmo AI, Ireland, and distributed by Medtronic, US), a CADe device for the detection of colorectal polyps that received marketing clearance in the United States from FDA in 2021^14^. The new device, named GI Genius v3.0, received CE clearance under the European Medical Device Directive (MDD, 93/42/EEC) in 2021 as a class IIa medical device. The performance assessment and results reported in this paper have been obtained using GI Genius v3.0 on a prospective dataset, acquired subsequently after approval and different from the dataset used during the development of the device, as described in the following paragraph in detail.

### Prospective dataset for performance testing: CHANGE study description

**Fig. 5:**
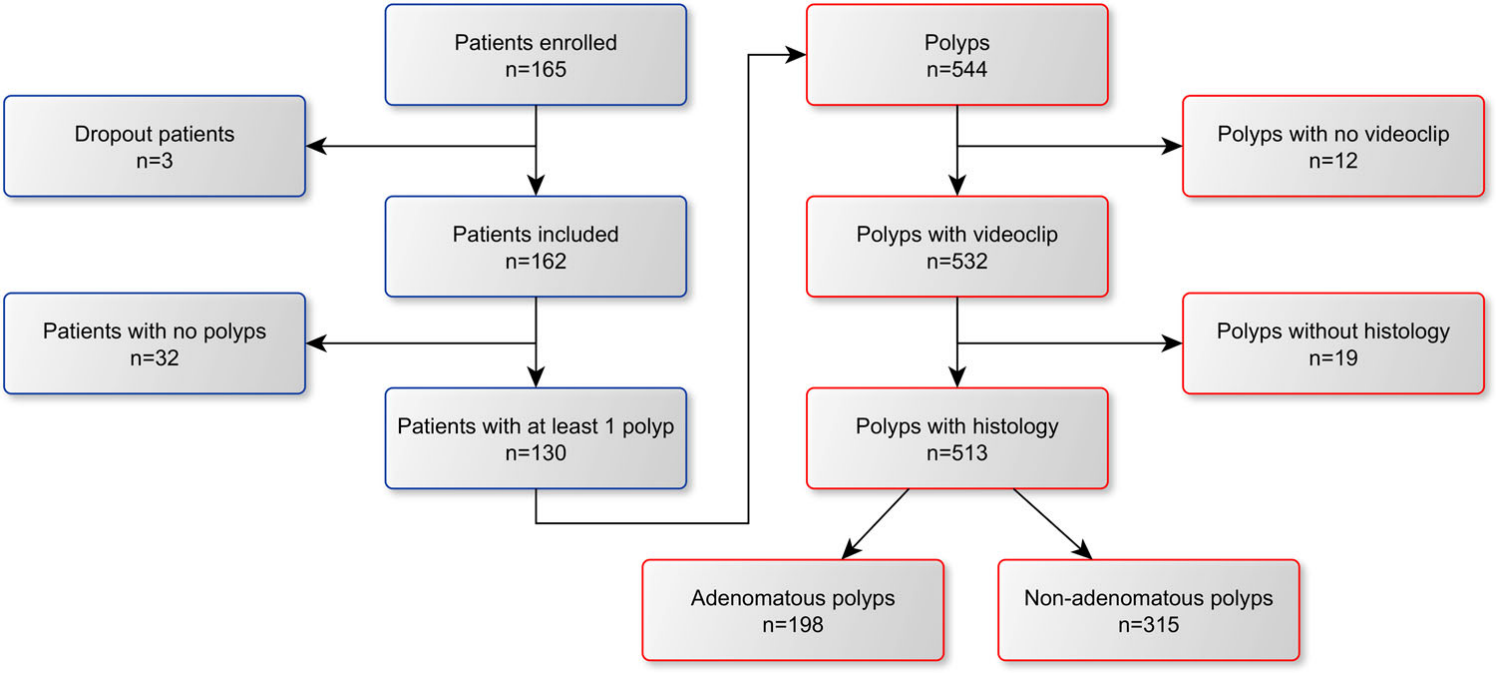
Colonoscopy videos of 165 patients screened in the CHANGE study were considered for the Standalone CADx study. A total of 544 polyps videoclips from 130 patients was obtained after discarding dropout patients and patients with no polyps. This number was further reduced to 513 polyp videoclips (198 adenomas, 315 non-adenomas) after polyps with no recorded histology or missing videoclips were discarded by scientific annotation experts.

The CHANGE study (“Characterization Helping in the Assessment of Neoplasia in Gastrointestinal Endoscopy”, ClinicalTrials.gov NCT04884581), a single-center, single-arm, prospective study acquired high-resolution videos of colonoscopy procedures conducted using GI Genius CADx v3.0 from May 2021 until July 2021. The study was approved by the local Institutional Review Board (Comitato Etico Lazio 1, prot. 611/CE Lazio 1) and conducted in accordance with the Declaration of Helsinki. Before participation, all participants provided written informed consent. The 165 patients screened in the CHANGE study were considered for the Standalone CADx study (“Standalone Performances of Artificial Intelligence CADx for Optical Characterization of Colorectal Polyps”, https://osf.io/m5cxt), a study aiming at assessing the standalone performance of the CADx and whose results are reported in this manuscript. A diagram illustrating the collection of the prospective dataset used in this study is shown in Fig. 5. All the colonoscopy videos considered in the study were acquired in full length with unaltered quality, bearing no trace of the AI used (no overlay). Patients' clinical data and polyp histopathological information were saved in an electronic Case Report Form (eCRF). The localization of each polyp in each patient was carefully annotated by scientific annotation experts. This was confronted with data in the eCRF for the same patient to avoid any possibility of erroneous correspondence between polyp in the video and the related histology. Polyps for which video recording failed or for which no histology could be obtained were excluded. For each polyp, a short videoclip was prepared, starting a few seconds before the first polyp appearance and ending with polyp endoscopic resection. If multiple polyps were present in the same video section, a separate clip was generated for each individual polyp. This resulted in a total of 513 videoclips, 198 adenomas and 315 non-adenomas.

### CHANGE study polyps review by endoscopists

To assess the performance of the CADx against a panel of international endoscopists, the study aimed at a minimum target of eight expert and eight non-expert endoscopist reviewers. Reviewers with a colonoscopy experience of at least 5 years and proficiency in optical biopsy with virtual chromoendoscopy were considered experts, while reviewers that had performed less than 500 colonoscopies at the time of study invitation were considered non-experts. To reach the target, 20 invitations were sent considering a 20% dropout. However, 10 additional invitations were needed and a final number of 10 experts and 11 non-experts reviewers was reached. Videos were shown in a randomized order to each endoscopist via a dedicated secure website. Endoscopists were blinded to histology and CADx results and a green box was manually drawn (overlaid) around the target polyp in each videoclip frame to remove any ambiguity in the identification of the region of interest.

### Measurement variables, study endpoints, and sample size considerations

The CADx decision could assume a three-classes output for each polyp videoclip: “adenoma”, “non-adenoma” and “undetermined”. A polyp was classified as “adenoma” if the number of frames where CADx outputs the label “adenoma” was greater than or equal to the number of frames where CADx outputs “non-adenoma”, and classified as “non-adenoma” if the number of frames classified as “non-adenoma” was greater than the number of frames classified as “adenoma”. A polyp was considered “undetermined” if the CADx failed to output either the label “adenoma” or “non-adenoma” for the entire polyp videoclip. A decision in WL and BL was retrieved by operating the CADx only on the frames in WL and BL, respectively.

Reviewers were asked to classify each polyp videoclip into five classes: “adenoma”, “hyperplastic”, “SSL”, “carcinoma” or “uncertain”. A reviewer decision was considered as “adenoma” if the reviewer selected either “adenoma” or “carcinoma”, “non-adenoma” if “hyperplastic” or “SSL” were selected and “undetermined” if “uncertain” was selected. If not “uncertain” the reviewer was asked a four-level level confidence class: “very high confidence”, “high confidence”, “low confidence” and “very low confidence”.

The primary endpoint of the Standalone CADx study was that CADx accuracy in WL resulted non-inferior to the accuracy of expert endoscopists, having histopathology as the reference standard. The exploratory endpoints were that (1) CADx accuracy in WL was superior to the accuracy of non-expert endoscopists and that (2) CADx accuracy in BL was non-inferior to CADx accuracy in WL.

A previous pilot study involving GI Genius CADx on 60 patients reported an accuracy of 85%. The sample size for Standalone CADx study was calculated assuming that experts can perform OC with an accuracy of 87%. Using a one-sided alpha level of 0.025, a total of 480 lesions is required to achieve 80% power, which is increased by 5% to account for dropouts. The minimum number of polyps needed for Standalone CADx study was therefore determined to be 504. Since CHANGE study collected a total of 513 polyps with a valid video recording and a valid histopathology outcome, all these polyps were included in the Standalone CADx statistical analysis.

### Statistical analysis

The analysis for the primary endpoint was to assess if the lower bound of 95% confidence interval (CI) for the difference in accuracies (CADx_WL_ – Experts) is higher than −10%. The analysis for the first exploratory endpoint was to assess if the lower bound of 95% CI for the difference in accuracies (CADx_WL_–Non-Experts) is greater than 0. The analysis for the second exploratory endpoint was to assess if the lower bound of 95% CI for the difference in accuracies (CADx_BL_–CADx_WL_) is greater than −10%. The main analysis of the primary and exploratory endpoints was carried out using log-binomial regression. As the primary and the first exploratory endpoints are involving repeated measures carried out by different readers, reader was considered as a random effect (random intercept) to account for intra-reader correlations.

A second analysis on performance comparisons was carried out using area under the receiver operating characteristic (ROC) curves. Both non-inferiority and superiority were evaluated using 95% two-sided CI calculated using bootstrap resampling for the paired difference in AUC. Success for non-inferiority was claimed when the lower bound of CI for the difference in AUCs was greater than –10%. Success for superiority was claimed when the lower bound of CI for the difference in AUCs was greater than 0. Non-expert and expert group ROC curves were obtained by transforming the survey confidence output assigned by each reviewer to each polyp into an eight-level score. CADx ROC curves were obtained by associating with each polyp the ratio between the number of polyp frames classified as “adenoma" and the number of frames on which a prediction was given by the CADx. For each bootstrap iteration, we randomly sampled with replacement all the 513 polyps to obtain new CADx ROC WL and BL curves, and subsequently, for the same set of sampled polyps, we randomly sampled at the reviewer level to compute expert and non-expert reviewers' ROC curves. We repeated this 10,000 times to define the 95% CI.

### Reporting summary

Further information on research design is available in the Nature Research Reporting Summary linked to this article.

## Electronic supplementary material

Supplementary Materials

Study protocol

Reporting Summary Checklist

Example Polyp 1

Example Polyp 2

Example Polyp 3

## Data Availability

De-identified study data may be made available at publication upon request to the corresponding author. Data sharing will only be available for academic research, instead of commercial use or other objectives. A data use agreement and institutional review board approval will be required as appropriate.

## Code availability

The underlying algorithm is copyrighted by Cosmo AI/Linkverse, and will not be available to the public. The authors agree to apply the algorithm to data provided by other academic researchers on their behalf for research purposes only.
