# Author Correction: A novel AI device for real-time optical characterization of colorectal polyps

**DOI:** 10.1038/s41746-022-00669-8

**Published:** 2022-08-16

**Authors:** Carlo Biffi, Pietro Salvagnini, Nhan Ngo Dinh, Cesare Hassan, Prateek Sharma, GI Genius CADx Study Group, Giulio Antonelli, Halim Awadie, Sebastian Bernhofer, Sabela Carballal, Mário Dinis-Ribeiro, Agnès Fernández-Clotet, Glòria Fernández Esparrach, Ian Gralnek, Yuta Higasa, Taku Hirabayashi, Tatsuki Hirai, Mineo Iwatate, Miki Kawano, Markus Mader, Andreas Maieron, Sebastian Mattes, Tastuya Nakai, Ingrid Ordas, Raquel Ortigão, Oswaldo Ortiz Zúñiga, Maria Pellisé, Cláudia Pinto, Florian Riedl, Ariadna Sánchez, Emanuel Steiner, Yukari Tanaka, Andrea Cherubini

**Affiliations:** 1 Artificial Intelligence Group, Cosmo AI/Linkverse, Lainate/Rome, Italy; 2 Department of Biomedical Sciences, Humanitas University, Via Rita Levi Montalcini 4, 20072 Pieve Emanuele, Milan Italy; 3 Endoscopy Unit, Humanitas Clinical and Research Center IRCCS, Rozzano, Italy; 4 VA Medical Center, Kansas City, MO USA; 5 University of Kansas School of Medicine, Kansas City, MO USA; 6 Milan Center for Neuroscience, University of Milano–Bicocca, 20126 Milano, Italy; 7 Gastroenterology and Digestive Endoscopy Unit, Ospedale dei Castelli (N.O.C.), Ariccia, Italy; 8 Gastrointestinal and Liver Institute, Emek Medical Center, Afula, Israel; 9 Gastroenterology and Hepatology and Rheumatology, University Hospital of St. Pölten, St. Pölten, Austria; 10 Gastroenterology Department, Hospital Clinic of Barcelona, Barcelona, Spain; 11 Gastroenterology Department, Portuguese Oncology Institute of Porto, Porto, Portugal; 12 Department of Gastroenterology, Kita-Harima Medical Center, Ono City, Japan; 13 Gastroenterology Department, Hyogo Cancer Center, Hyogo, Japan; 14 Gastroenterology Department, Sugita Genpaku Memorial Obama Municipal Hospital, Obama, Japan; 15 Gastrointestinal Center, Sano Hospital, Hyogo, Japan; 16 Kobe Red Cross Hospital, Hyogo, Japan; 17 Kobe University Hospital, Hyogo, Japan

**Keywords:** Colonoscopy, Image processing

Correction to: *npj Digital Medicine* 10.1038/s41746-022-00633-6, published online 30 June 2022

In this article the affiliation details for Cesare Hassan were incorrectly given as ‘Gastroenterology Unit, Nuovo Regina Margherita Hospital, Rome, Italy’ but should have been ‘Department of Biomedical Sciences, Humanitas University, Via Rita Levi Montalcini 4, 20072 Pieve Emanuele, Milan, Italy’. The original article has been corrected.

