# Supplementary material for: A novel AI device for real-time optical characterization of colorectal polyps
Source: NPJ Digit Med. 2022 Jun 30;5:84. doi: 10.1038/s41746-022-00633-6 (PMC9247164; doi:10.1038/s41746-022-00633-6)
Supplement: Supplementary file 1 — Supplementary Materials [file 41746_2022_633_MOESM1_ESM.pdf]

A novel AI device for real-time optical characterization of colorectal polyps

Supplementary Materials

Supplementary Note 1: Additional information on the AI device processing times

The proposed AI medical device operates with a direct video transmission configuration. According to this configuration, the original frame received in input is directly forwarded to the output with a minimum delay ($\leq 1.5\ \mu$s). The same frame is processed on a parallel path by the device algorithms, with the results of the processing provided as an overlay. Since the sum of all transfer, synchronization and processing times is between 50 and 60 ms, the overlay resulting from processing of the frame is displayed on a frame which is after the one used for processing. This lag was measured quantitatively within the device validation by submitting a video with a content known to be detected by the CAD, counting the frames needed to see the activation overlay and calculating the time by multiplying this value to the frame hanging time. It is worth adding that, to the lag described above, there is no associated frame drop by design – the device is continuously processing all the frames coming in, as none of the single processing tasks forming the processing pipeline is taking longer than the frame hanging time.

Supplementary Note 2: Additional information on lossless video recording for the CHANGE study

CHANGE study full procedure videos were recorded with a professional video recorder (Atomos Shogun Inferno) able to record YUV video stream with no loss of color and resolution: 4:2:2 chroma subsampling and 10-bit per sample at the resolution of 1920x1080 (interlaced), as the original stream. Recorded videos were compressed with Apple ProRes high quality codec, with an average file size of 30 GB and an average duration of 20:33 minutes.

Supplementary Note 3: Video interface for polyp videoclip assessment

In Fig. 1 we report a screenshot of the web video interface used by endoscopist reviewers to assess the polyp videoclips from the prospective dataset acquired for this study. The interface allowed the user to fully control what to play, allowing the endoscopist to replay, pause, skip video parts etc. so as to fully evaluate the imaged polyp with no time limitations.

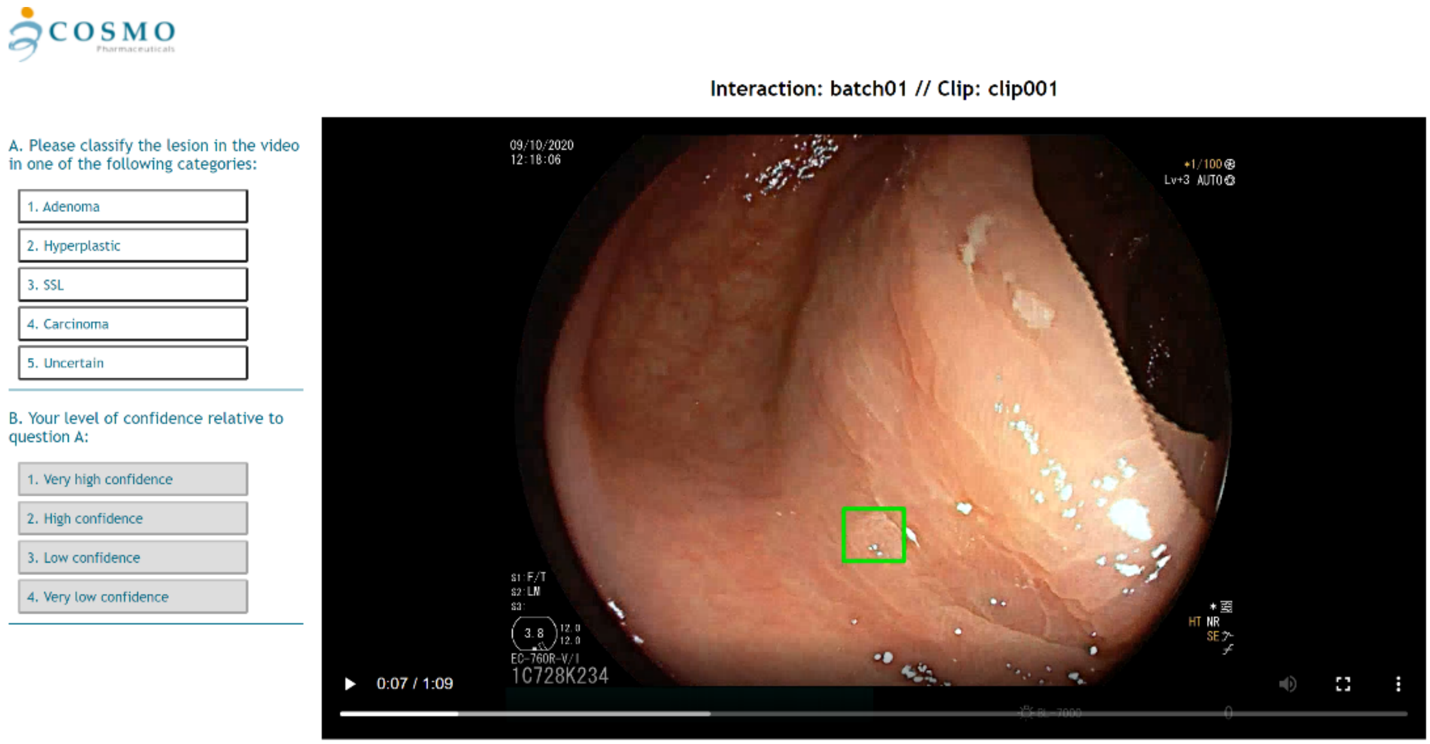

Supplementary Figure 1: **Screenshot of the online survey web interface.**

The review of the polyps of the prospective study was run in parallel to the acquisition of the patients in the CHANGE study. Every 84 consecutive polyps of the CHANGE study were considered as an independent batch and sent for reviewers' assessment. For each batch, 20 pre-randomization lists for the order of presentation of polyps - one for each reviewer - were prepared. Each endoscopist reviewer was preassigned a pre-randomization ID, and polyps in each batch were presented to the reviewer with an order corresponding to the pre-randomization ID. Data was collected by means of an online survey for which each participant was provided with a unique username and password. Reviewers who completed all required evaluations were acknowledged in the GI Genius CADx Study Group.

For each polyp, endoscopist reviewers were asked to answer the following questions:

- Please classify the polyp in the video in one of the following categories: (Adenoma / Hyperplastic / SSL / Carcinoma / Uncertain)
- Your level of confidence relative to previous question: (Very high confidence / High confidence / Low confidence / Very low confidence)

Question about confidence was offered only in case the participant did not choose "Uncertain" in the first question. All response times were also logged. It was not possible to change the answer to one question once it was recorded. We stress the fact that all endoscopist reviewer answers regarding the polyp optical characterization were decided solely according to their clinical experience, without any CADx overlay on the video and without any histological information.

Supplementary Results 1: Descriptive statistics on label timings

The 513 polyps video clip duration was (mean±standard deviation): 91.1s±56.6s. Overall, there were 677985 frames with a CADe detection, 480830 in white light (70.9%) and 197155 in blu light (29.1%). The time needed to output the first label for a polyp was 5.81s±2.61s. The proportion of adenomas between the polyps that the CADx characterized faster (first label output less than 5.81s; 50.9% of the total) was 41.9%, to be compared with 36.6% in the "slower" group (first label output greater or equal than 5.81s; 49.1% of the total). No-prediction was displayed at least once in 386 polyps (75.2%)

Supplementary Results 2: Additional data on polyps

Anatomical location	Polyps
caecum	39
ascending	70
hepatic flexure	4
transverse	51
splenic flexure	1
descending	41
sigma	161
rectum	146

Supplementary Table 1: **Distribution of polyps according to anatomical location.**

Size [mm]	Polyps
1	158
2	181
3	60
4	43
5	23
6	11
7	5
8	2
10	11
13	1
15	13
20	5

Supplementary Table 2: **Distribution of polyps according to estimated size.**

Paris class - Description	Polyps
Ip - Pedunculated	8
Is - Sessile	19
Isp - Subpedunculated	3
IIa - Flat elevated	469
IIb - Completely flat	14

Supplementary Table 3: **Distribution of polyps according to the Paris classification of morphology.**

Histology	Polyps
hyperplastic	259
sessile serrated lesion	10
TSA	3
no-polyp	43
Total non-adenomatous	315
tubular adenoma	178
tubulovillous adenoma	20
Total adenomatous	198

Supplementary Table 4: **Distribution of polyps according to histology.**

Number of switches	Number of Polyps
0	396
1	49
2	34
3	7
4	7
5	1
undetermined	19

Supplementary Table 5: **Number of "adenoma" and "non-adenoma" switches.** Number of CADx switches between "adenoma" and "non-adenoma" frame-by-frame output during the optical characterization of the CHANGE dataset polyps. 0 switches indicates that the CADx did not change between "adenoma" and "non-adenoma" during the time a single polyp was visible. Undetermined indicates that the CADx failed to output neither of the label "adenoma" nor "non-adenoma" during the time a single polyp was visible.

Supplementary Results 3: Additional exploratory analyses

Two additional exploratory analyses were performed to assess the robustness of the results to a different categorization of lesion histology and sizing. These analyses were performed following the same statistical plan of the study.

Analysis on diminutive polyps

This analysis was performed only on the 465 diminutive polyps (5mm or less) in the dataset.

All the three endpoints were confirmed at the re-analysis, see below:

- CADx accuracy in WL was found to be non-inferior to the accuracy of Expert endoscopists ($CADx_{WL}$/Expert[illegible] OR 1.263 [0.790-2.018]; p<0.001)
- CADx accuracy in WL was found superior to the accuracy of Non-expert endoscopists ($CADx_{WL}$/Non-experts; OR 1.928 [1.187-3.130]; p=0.004)
- CADx accuracy in blue light was found non-inferior to CADx accuracy in WL ($CADx_{BL}$/$CADx_{WL}$; OR 0.864 [0.585-1.277]; p=0.006)

Analysis with different ground truth

This analysis was performed considering the 10 SSLs and 3 TSAs in the dataset as having a ground truth of "adenomas".

All the three endpoints were confirmed at the re-analysis, see below:

- CADx accuracy in WL was found to be non-inferior to the accuracy of Expert endoscopists ($CADx_{WL}$/Expert[illegible] OR 1.696 [1.135-2.535]; p<0.001)
- CADx accuracy in WL was found superior to the accuracy of Non-expert endoscopists ($CADx_{WL}$/Non-experts; OR 2.163 [1.595-2.934]; p<0.001)
- CADx accuracy in blue light was found non-inferior to CADx accuracy in WL ($CADx_{BL}$/$CADx_{WL}$; OR 0.846 [0.590-1.213]; p=0.007)

Supplementary Results 4: Individual reviewer performances table

	Accuracy	Sensitivity	Specificity	No prediction
expert01	74,1 % [70,1-77,7]	81,6 % [75,6-86,5]	69,3 % [63,9-74,1]	1,6 % [0,8-3,1]
expert02	83,3 % [79,8-86,3]	72,1 % [65,4-77,9]	90,4 % [86,6-93,2]	0,6 % [0,2-1,8]
expert03	82,5 % [78,9-85,7]	73,1 % [66,4-78,8]	88,9 % [84,7-92,0]	6,2 % [4,4-8,7]
expert04	80,9 % [77,3-84,1]	69,7 % [62,9-75,7]	87,9 % [83,9-91,1]	0,0 % [-]
expert05	83,2 % [79,6-86,2]	66,5 % [59,3-73,0]	92,9 % [89,4-95,3]	5,1 % [3,5-7,3]
expert06	86,2 % [83,0-89,0]	83,3 % [77,5-87,9]	88,1 % [84,0-91,3]	0,8 % [0,3-2,1]
expert07	82,7 % [79,1-85,7]	72,7 % [66,1-78,5]	88,9 % [84,9-91,9]	0,0 % [-]
expert08	78,5 % [74,7-81,8]	77,8 % [71,5-83,0]	78,9 % [74,0-83,1]	0,4 % [0,1-1,5]
expert09	83,2 % [79,8-86,2]	82,3 % [76,4-87,0]	83,8 % [79,3-87,5]	0,0 % [-]
expert10	86,2 % [82,9-88,9]	83,3 % [77,5-87,9]	87,9 % [83,9-91,1]	0,0 % [-]
non-expert01	76,8 % [72,9-80,3]	61,2 % [54,0-67,9]	86,5 % [82,2-89,9]	4,1 % [2,7-6,2]
non-expert02	76,8 % [72,9-80,2]	67,7 % [60,9-73,8]	82,5 % [77,9-86,3]	0,2 % [0,0-1,4]
non-expert03	74,2 % [70,3-77,8]	52,3 % [45,3-59,2]	87,9 % [83,9-91,1]	0,2 % [0,0-1,4]
non-expert04	79,0 % [75,1-82,5]	55,1 % [47,7-62,3]	93,2 % [89,8-95,6]	8,0 % [5,9-10,7]
non-expert05	65,8 % [61,6-69,8]	90,9 % [86,0-94,2]	50,0 % [44,5-55,5]	0,2 % [0,0-1,4]
non-expert06	78,6 % [74,8-81,9]	78,8 % [72,5-83,9]	78,4 % [73,5-82,6]	0,0 % [-]
non-expert07	70,4 % [66,3-74,2]	48,2 % [41,3-55,2]	84,5 % [80,1-88,1]	1,2 % [0,5-2,6]
non-expert08	77,3 % [73,4-80,7]	72,4 % [65,8-78,2]	80,3 % [75,5-84,3]	0,6 % [0,2-1,8]
non-expert09	74,1 % [70,1-77,8]	65,1 % [58,1-71,5]	79,7 % [74,9-83,9]	2,9 % [1,8-4,8]
non-expert10	70,1 % [66,0-74,0]	97,4 % [94,0-98,9]	52,8 % [47,2-58,3]	2,1 % [1,2-3,8]
non-expert11	79,6 % [75,9-82,9]	65,7 % [58,8-71,9]	88,5 % [84,4-91,6]	0,6 % [0,2-1,8]

Supplementary Table 6: **Performance metrics for individual reviewers.** Accuracy, Sensitivity and Specificity are calculated on the fraction of 513 polyps for which a prediction was provided.

GI Genius CADx Study Group

Giulio Antonelli[a], Halim Awadie[b], Sebastian Bernhofer[c], Sabela Carballal[d], Mário Dinis-Ribeiro[e], Agnès Fernández-Clotet[d], Glòria Fernández Esparrach[d], Ian Gralnek[b], Yuta Higasa[f], Taku Hirabayashi[g], Tatsuki Hirai[h], Mineo Iwatate[i], Miki Kawano[j], Markus Mader[c], Andreas Maieron[c], Sebastian Mattes[c], Tastuya Nakai[k], Ingrid Ordas[d], Raquel Ortigão[e], Oswaldo Ortiz Zúñiga[d], Maria Pellisé[d], Cláudia Pinto[e], Florian Riedl[c], Ariadna Sánchez[d], Emanuel Steiner[c], Yukari Tanaka[g]

Affiliations

[a] *Gastroenterology and Digestive Endoscopy Unit, Ospedale dei Castelli (N.O.C.), Ariccia, Italy*

[b] *Gastrointestinal and Liver Institute, Emek Medical Center, Afula, Israel*

[c] *Gastroenterology and Hepatology and Rheumatology, University Hospital of St. Pölten, Austria*

[d] *Gastroenterology Department, Hospital Clinic of Barcelona, Barcelona, Spain*

[e] *Gastroenterology Department, Portuguese Oncology Institute of Porto, Portugal*

[f] *Department of Gastroenterology, Kita-Harima Medical Center, Ono city, Japan*

[g] *Gastroenterology Department, Hyogo Cancer Center, Hyogo, Japan*

[h] *Gastroenterology, Department, Sugita Genpaku Memorial Obama Municipal Hospital, Obama, Japan*

[i] *Gastrointestinal Center, Sano Hospital, Hyogo, Japan*

[j] *Kobe Red Cross Hospital, Hyogo, Japan*

[k] *Kobe University Hospital, Hyogo, Japan*
