# Supplementary material for: A novel AI device for real-time optical characterization of colorectal polyps
Source: NPJ Digit Med. 2022 Jun 30;5:84. doi: 10.1038/s41746-022-00633-6 (PMC9247164; doi:10.1038/s41746-022-00633-6)
Supplement: Supplementary file 2 — Study protocol [file 41746_2022_633_MOESM2_ESM.pdf]

STUDY PROTOCOL SYNOPSIS

Title of Study: Standalone Performances of Artificial Intelligence CADx for Optical Characterization of Colorectal Polyps

Study code: Standalone CADx

Guideline: This study protocol is designed according to SPIRIT-AI guidelines SPIRIT-AI

Study design: Single-center, single-arm, prospective study

Investigational Medical Device (IMD): GI Genius CADx, software version 3.0.0

Study Registration: clinical data and endoscopy procedures used for present study are prospectively acquired according to study CHANGE (Characterization Helping the Assessment of Colorectal Neoplasia in Gastrointestinal Endoscopy, Clinicaltrials.gov registration NCT04884581)
This document detailing aims and methods for Standalone CADx sub-study is registered on osf.io

Protocol version: version 1.0 – 15/06/2021

Funding: this is a post-marketing study sponsored by Cosmo AI, part of Cosmo Pharmaceuticals

Roles and responsibilities:
Protocol contributors:
Andrea Cherubini, PhD – Head of Artificial Intelligence – Linkverse Srl – Cosmo Pharmaceuticals, Lainate (MI), Italy
Cesare Hassan, MD – P.I. of CHANGE Study, Ospedale Nuovo Regina Margherita, Rome, Italy
Study Roles:
Cesare Hassan, MD: CHANGE study design; collection, management of data; Standalone CADx study design; writing of the report
Andrea Cherubini, PhD: GI Genius CADx development; CHANGE and Standalone CADx study design; analysis and interpretation of data; endpoint adjudication; writing of the report
Pietro Salvagnini, PhD: GI Genius CADx development; analysis and interpretation of data; writing of the report
Carlo Biffi, PhD: GI Genius CADx development; analysis and interpretation of data; writing of the report
Sponsor Role:
Standalone CADx (AI data analysis) is a post-marketing study sponsored by Cosmo AI whose aim is to assess the performances of GI Genius CADx, developed by Cosmo AI; Andrea Cherubini, Pietro Salvagnini and Carlo Biffi are employees of Linkverse Srl – Cosmo AI, part of Cosmo Pharmaceuticals
CHANGE study (independent prospective data collection) is an investigator-initiated study, not sponsored by Cosmo AI

Background and Rationale:

Real-time in vivo Optical Characterization (OC) of colorectal polyps during colonoscopy has recently gained traction as means to guide 'resect or discard' strategies, potentially leading to decreased complications, physician burden, and medical costs. However, this characterization task is challenging and suffers from significant inter- and intra-observer variability, often resulting in community endoscopists performances below accepted thresholds. Several learning-based models have been recently proposed to aid this characterization task. Still, none of them can work on real-time regular colonoscopy videos, as they have either been developed for the characterization of still images or they require human intervention to cope with variations of image quality and polyp appearance.
In this study, we intend to assess the performances of a deep learning-based module for real-time characterization of colorectal polyp's histology that can be coupled with any automated polyp detection model in colonoscopy videos (GI Genius CADx). The module consists of a convolutional neural network model which classifies each detected polyp in a single video frame as adenomatous or non-adenomatous polyp and tracks it across all the video frames. This algorithm produces stable, spatio-temporally weighted decisions that are displayed real-time on each frame. The AI model can also abstain from predicting the polyp histology if insufficient confidence was accumulated. The GI Genius CADx was developed to help endoscopists in their clinical practices for polyp characterization.

In order to assess GI Genius CADx performances in terms of prediction accuracy, the output of GI Genius CADx (adenoma/non-adenoma/no-prediction) will be evaluated against the histopathology reference standard (ground truth). The performances of GI Genius CADx will be compared against the prediction of the same lesions performed by a pool of endoscopists reviewing the video recording of the procedures blind to the histology results.

Objective:

To prospectively evaluate if GI Genius CADx accuracy in the automated OC of colorectal polyps in white light is non-inferior to the accuracy of expert endoscopists performing OC (supported by virtual chromoendoscopy), having histopathology as a reference standard.
Furthermore, to evaluate if GI Genius CADx accuracy in the automated OC of colorectal polyps in white light is superior to the accuracy of non-expert endoscopists performing OC (supported by virtual chromoendoscopy), having histopathology as a reference standard.
Furthermore, to evaluate if GI Genius CADx accuracy in the automated OC of colorectal polyps in white light is non-inferior to GI Genius CADx accuracy in virtual chromoendoscopy, having histopathology as a reference standard.

Study sites:

Colorectal polyps will be prospectively collected at Ospedale Nuovo Regina Margherita, Rome, Italy

Subject population:

Patients aged 40-80 undergoing colonoscopy

Endoscopist reviewers invitation:

After Standalone CADx protocol registration, we will send invitations to expert and non-expert endoscopist reviewers who will be asked to perform optical characterization of video recordings of lesions collected in CHANGE study.
Experts are endoscopists having more than 5 years of colonoscopy experience, a proven track of scientific publications on optical characterization or a similar subject, and experience in optical biopsy with virtual chromoendoscopy. An endoscopist is considered "non-expert" if s/he has performed less than 500 colonoscopies.
Our minimum target number of reviewers completing the whole set of stimuli is 16: 8 experts, and 8 non-experts. To reach the target in each subgroup we will send 20 invitations in the first wave, considering a 20% dropout. If less than 8 + 8 reviewers agreed to participate in the study after two weeks, a second wave of 10 invitations will be sent (weighted for the proportion of missing reviewers in the two main classes). The same criterion and waiting time will be applied to the possible successive invitation waves. Once the targeted participant pool has been reached, we will stop invitations.
After the data acquisition has started, we will monitor for potential dropouts. Whenever the total active reviewers fall below the minimum targeted values (8+8) we will resume invitation with a ratio of 2:1, i.e., two invitations for each participant missing to the target. Thus, if we find ourselves with 7 non-expert participants because of dropouts, we will invite another 2.
The data collection for Standalone CADx study will be closed when at least 8 non-expert and 8 expert endoscopists will have completed all required evaluations.
All data will be used in the analyses, even if data collection produced slightly more data than the minimum target.

Eligibility criteria
Inclusion/Exclusion criteria for patients:
Inclusion criteria:

- Patients aged 40 years or older undergoing screening colonoscopy for CRC
- Ability to provide written, informed consent (approved by EC) and understand the responsibilities of trial participation.

Exclusion criteria:

- subject at high risk for CRC;
- subjects with a personal history of CRC, IBD or hereditary polyposis or non-polyposis syndromes;
- patients with previous resection of the sigmoid rectum;
- patients on anticoagulant therapy, which precludes resection / removal operations due to histopathological findings;
- patients who perform an emergency colonoscopy.

Inclusion/Exclusion criteria for lesions:
Inclusion criteria:

- First valid 504 lesions resected in the CHANGE study will be included in the Standalone CADx study, corresponding to six batches of 84 lesions each

Exclusion criteria:

- Lesions with technical problems in video recording
- Lesions without a corresponding histology outcome
- Samplings of tissues not corresponding to a colon lesion (e.g. normal colon mucosa, inflammatory tissue, etc.)

Study procedure:

Lesion collection

Lesions used in the present study will be prospectively acquired in the clinical study named CHANGE. Patients enrolled will undergo a standard white-light colonoscopy with the support of the latest version of the CE-marked GIG CADx device (version 3.0.0). Colonoscopy will be performed according to the standard practice. The primary endpoint of CHANGE study is to measure the Negative Predictive Value (NPV) of GI Genius CADx optical diagnosis on diminutive (≤5 mm) rectosigmoid polyps. CHANGE study estimated enrolment is of 156 participants, corresponding to a total of about 500-800 lesions. First valid 504 lesions resected in the CHANGE study will be included in the Standalone CADx study.

Electronic data recording

All endoscopy procedures in the CHANGE study will be recorded lossless (without image degradation or compression) and without any of the GI Genius markings from beginning to the end of the procedure. In other words, the video of the recorded procedures will bear no trace of the AI used. All clinical data of the participants and of the retrieved lesion will be recorded in an electronic CRF using openclinica (www.openclinica.com).

Batch subdivision

For the Standalone CADx study, the following process will be used for video preparation. Every 84 consecutive lesions will be considered as an independent batch. Batches will be numbered in progressive order: batch01, batch02, etc. As soon as a batch is completed all video recordings in the batch will be prepared for lesion review. With this strategy, the review of the lesions via the website needed for the present study will be run in parallel to the acquisition of the patient in the CHANGE study.

Videoclip check and annotation

All video procedures from a given batch will be carefully examined by scientific annotator experts in the task of video preparation for artificial intelligence elaboration. In particular, the timing and the localization of each lesion in each patient of the batch will be carefully annotated and confronted with data of the lesion in the eCRF for the same patient, to avoid any possibility of erroneous correspondence between lesion in the video and following histology outcome. Lesions for which no recording is available will be excluded from Standalone CADx study.

For each lesion, a short video clip will be prepared, starting a few seconds before the first lesion appearance and ending with lesion surgical resection. If multiple lesions are present in the same video section, a separate clip will be generated for each individual lesion. During the first ten seconds of the videoclip, a green box will be manually drawn (overlaid) around the target lesion. There will be no other graphical marks except for this green box indicating the lesion position. The purpose of this marking is to avoid to the endoscopist reviewing the clip any ambiguity in identifying which is the region of interest about which it is asked to express a prediction of the histology. To be noted that the markings is not generated by AI, and its only purpose is to clearly identify lesion to be assessed. Consequently, in Standalone CADx study there is no interaction between AI-generated content and humans.

Optical characterization of lesions by endoscopists reviewers

After the procedure has been carried out and data relative to colonoscopy has been properly recorded in eCRF, before the availability of the histology results, without the presence of the patient and the endoscopist who has performed the procedure, the video recording will be reviewed by a team of endoscopists reviewers to get their optical biopsies and confidence about polyp adenomatous or non-adenomatous histology.

Randomization

Overall, we will use in Standalone CADx study 6 batches with 84 lesions each, for a total of 504 lesions. Possible further lesions that will be available from the CHANGE study are not going to be used in the present experiment. For each batch, 20 pre-randomization lists will be prepared for the order of presentation of lesions. Each endoscopist reviewer will be preassigned a pre-randomization ID, and lesions in each batch will be presented to the reviewer with the order corresponding to the pre-randomization ID.

Online survey

Data will be collected by means of an online survey. Each participant will be provided with a unique username. The video interface (see figure above) will allow the user to fully control what to play, allowing the endoscopist to skip, view again, pause, etc. We will record the viewing time for each frame.

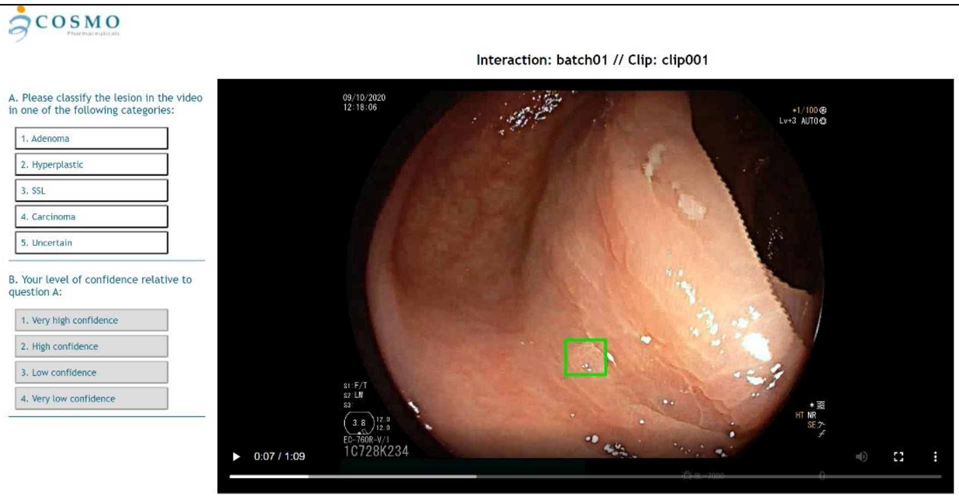

The endoscopist reviewers need to answer the following questions:

A. Please classify the lesion in the video in one of the following categories:
1. Adenoma
2. Hyperplastic
3. SSL
4. Carcinoma
5. Uncertain

B. Your level of confidence relative to question A:
1. Very high confidence
2. High confidence
3. Low confidence
4. Very low confidence

Question B will be offered only in case the participant does not choose “Uncertain” in question A.
All response times will be logged. It will not be possible to change the answer to one question once it has been recorded.
According to the study procedure described above there is no interaction between AI-generated content and humans. Consequently, all endoscopist reviewer answers regarding the lesion optical characterization are decided solely according to their clinical experience, without any AI output.
The data collection for Standalone CADx study via the online survey will be closed when at least 8 non-expert and 8 expert endoscopists will have completed all required evaluations.

Measurement variables:

GI Genius CADx prediction: the diagnosis about a given lesion provided by GI Genius. It takes the following values: “adenoma”, “non-adenoma”, and “undetermined”. The output of GI Genius CADx for a given lesion is a time series with a unique value for each image frame, starting with the first GI Genius CADe detection of the lesion and terminating at the last image frame of the video clip, when the lesion is resected. Of all time frames when the lesion is visible, only those where GI Genius CADx has expressed one of the possible three labels are considered: “adenoma” / “non-adenoma” / “no-prediction”. The GI Genius CADx prediction will be considered “adenoma” if the number of frames where GI Genius CADx outputs the label “adenoma” is greater than or equal to the number of frames where GI Genius CADx outputs “non-adenoma”. The GI Genius CADx prediction will be considered “non-adenoma” if the number of frames where GI Genius CADx outputs the label “non-adenoma” is greater than the number of frames where GI Genius CADx outputs “adenoma”. If GI Genius CADx fails to output either the label “adenoma” or “non-adenoma” for the entire clip of a given lesion, the GI Genius CADx prediction will be considered “undetermined”.

GI Genius CADx prediction in white light: the GI Genius CADx prediction according to the method described above, considering only the frames in white light (the subset of the lesion frame timeseries in white light).

GI Genius CADx prediction in blu light: the GI Genius CADx prediction according to the method described above, considering only the frames in blu light (the subset of the lesion frame timeseries when virtual chromoendoscopy is activated).

GI Genius CADx confidence: the degree of certainty about the response (prediction) given by GI Genius CADx. It takes the following values: “high confidence”, “low confidence”, and “undetermined”. GI Genius CADx confidence will be considered “undetermined” if the GI Genius CADx prediction is “undetermined”. GI Genius CADx confidence will be considered “low confidence” if the number of frames where GI Genius CADx outputs the label “no-prediction” is greater than or equal to the sum of the number of frames where GI Genius CADx outputs “adenoma” and the number of frames where GI Genius CADx outputs “non-adenoma”. GI Genius CADx confidence will be considered “high confidence” if the number of frames where GI Genius CADx outputs the label

"no-prediction" is smaller than the sum of the number of frames where GI Genius CADx outputs "adenoma" and the number of frames where GI Genius CADx outputs "non-adenoma".

Endoscopist reviewer prediction: the diagnosis about a given lesion provided by a reviewer. It takes the following values: "adenoma", "non-adenoma", and "undetermined". The endoscopist reviewer prediction for a specific lesion will be considered "adenoma" if the corresponding answer to the question A of the online survey is "1. Adenoma" or "4. Carcinoma". The endoscopist reviewer prediction for a specific lesion will be considered "non-adenoma" if the corresponding answer to the question A of the online survey is "2. Hyperplastic" or "3. SSL". The endoscopist reviewer prediction for a specific lesion will be considered "undetermined" if the corresponding answer to the question 1 of the online survey is "5. Uncertain".

Endoscopist reviewer confidence: the degree of certainty about the response (prediction) provided about a given lesion. It takes the following values: "high confidence", "low confidence", and "undetermined". The endoscopist reviewer confidence for a specific lesion will be considered "high confidence" if the corresponding answer to the question B of the online survey is "1. Very high confidence" or "2. High confidence". The endoscopist reviewer confidence for a specific lesion will be considered "low confidence" if the corresponding answer to the question B of the online survey is "3. Low confidence" or "4. Very low confidence". The endoscopist reviewer confidence for a specific lesion will be considered "undetermined" if the corresponding answer to the question A of the online survey is "5. Uncertain".

Histology ground truth: the histopathological evaluation provided about a given lesion. It takes the following values: "adenoma", and "non-adenoma". The histopathological evaluation will be based on the revised Vienna classification of gastrointestinal epithelial neoplasia. Lesions corresponding to Vienna category 1 (negative for neoplasia) or 2 (indefinite for neoplasia) will be considered "non-adenoma". Lesions corresponding to Vienna category 3 (mucosal low-grade neoplasia), 4 (mucosal high-grade neoplasia) or 5 (submucosal invasion of neoplasia), will be considered "adenoma".

Study endpoints:

Primary endpoint:

1. GI Genius CADx accuracy in white light is non-inferior to the accuracy of expert endoscopists, having histopathology as a reference standard.

Exploratory endpoints:

1. GI Genius CADx accuracy in white light is superior to the accuracy of non-expert endoscopists, having histopathology as a reference standard.
2. GI Genius CADx accuracy in white light is non-inferior to GI Genius CADx accuracy in blu light (virtual chromoendoscopy), having histopathology as a reference standard.

Analysis plan:

1. Test for non-inferiority of GI Genius CADx accuracy in white light against accuracy of expert endoscopists supported by virtual chromoendoscopy, having histopathology as a reference standard, with a margin of 10%.
2. Test for superiority of GI Genius CADx accuracy in white light against accuracy of non-expert endoscopists supported by virtual chromoendoscopy, having histopathology as a reference standard.
3. Test for non-inferiority of GI Genius CADx accuracy in white light against GI Genius CADx accuracy in virtual chromoendoscopy, having histopathology as a reference standard.
4. Report descriptive analysis of Sensitivity, Specificity, Accuracy, of GI Genius CADx optical characterization for different subdivision of lesion according to size, anatomical location, endoscope, age, sex, reason for colonoscopy.

Sample size:

A previous pilot study involving GI Genius CADx on 60 patients, reported an accuracy of 85%. The sample size calculation is calculated assuming that experts can perform optical characterization with an accuracy of 87%. Using a one-sided alpha level of 0.025, a total of 480 lesions is required to achieve 80% power, which is increased by 5% to account for dropouts. The number of lesions for Standalone CADx study will therefore be 504.
CHANGE study plans to recruit 156 patients, which should correspond to about 500-800 lesions. Therefore, the first 504 lesions resected in CHANGE study, complete of a valid video recording and a valid histopathology outcome will be used for Standalone CADx study.

Timeline:

CHANGE study
Study registration on clinicaltrials.gov: May 12, 2021
First patient recruited: May 12, 2021
End of patient recruitment: July 2021
Standalone CADx study:
Study registration on osf.io: June 18, 2021
Email sent to first reviewers: June 21, 2021
End of study: September 2021

CLINICAL STUDY PROTOCOL

The CHANGE Study:
Characterization Helping the Assessment of colorectal Neoplasia in Gastrointestinal Endoscopy

PRINCIPAL INVESTIGATOR: Dott. Cesare Hassan, Nuovo Regina Margherita Hospital, Rome, Italy

STUDY COORDINATOR SIGNATURE

Protocol Title: CHANGE – Characterization Helping the Assessment of colorectal Neoplasia in Gastrointestinal Endoscopy

Protocol Number: CHANGE 1.5_08/04/2021

Study Sponsor: Dott. Cesare Hassan
Nuovo Regina Margherita Hospital
Rome, Italy

I have read the attached document, concur that it contains all information necessary to conduct the study, and agree to abide by all provisions set forth.
I agree to conduct the study in accordance with:

- ISO 14155 (ANSI AAMI ISO 14155:2011)
- Declaration of Helsinki
- Applicable national legal and regulatory requirements and national regulations for medical devices

I will not initiate the study until I have obtained approval by the appropriate Institutional Review Board or Ethic Committee and have complied with all financial and administrative requirements of the governing body of the clinical institution and the Sponsor. I will obtain written informed consent from each study patient prior to performing any study specific procedures.
I understand that my signature on a case report form (electronic or paper) indicates that the data therein has been reviewed and accepted by me.
The information contained in this protocol is proprietary and provided to me in confidence, and may not be disclosed to any other party, in any form, without prior authorization from the Sponsor except to the extent necessary for the conduct of the study at this study site.

____

Study Coordinator - Signature Date

____

Study Coordinator - Printed name

____

Institution name and address

INVESTIGATORS SIGNATURE PAGE

Protocol Title: CHANGE – Characterization Helping the Assessment of colorectal Neoplasia in Gastrointestinal Endoscopy

Protocol Number: CHANGE 1.5

Study Sponsor: Dott. Cesare Hassan
Nuovo Regina Margherita Hospital
Rome, Italy

I have read the attached document, concur that it contains all information necessary to conduct the study, and agree to abide by all provisions set forth.
I agree to conduct the study in accordance with:

- ISO 14155 (ANSI AAMI ISO 14155:2011)
- Declaration of Helsinki
- Applicable national legal and regulatory requirements and national regulations for medical devices

I will not initiate the study until I have obtained approval by the appropriate Institutional Review Board or Ethic Committee and have complied with all financial and administrative requirements of the governing body of the clinical institution and the Sponsor. I will obtain written informed consent from each study patient prior to performing any study specific procedures.
I understand that my signature on a case report form (electronic or paper) indicates that the data therein has been reviewed and accepted by me.
The information contained in this protocol is proprietary and provided to me in confidence, and may not be disclosed to any other party, in any form, without prior authorization from the Sponsor except to the extent necessary for the conduct of the study at this study site.

____
Principal Investigator - Signature   Date

____
Principal Investigator - Printed name

____
Institution name and address

STUDY SYNOPSIS

**Sponsor:** Dott. Cesare Hassan - Ospedale Nuovo Regina Margherita
**1. Title of Study:** CHANGE – Characterization Helping the Assessment of colorectal Neoplasia in Gastrointestinal Endoscopy
**2. Study code:** CHANGE
**3. Clinical phase:** post-marketing
**4. Study design:** Multi-center, single-arm, prospective study
**Investigational Medical Device (IMD):** GI Genius CADx, software version 3.0.0
**5. Background and Rationale:**<br><br>Diminutive colorectal polyps (≤ 5 mm) represent most of the polyps detected during colonoscopy, especially in the rectum-sigmoid tract. The characterization of these polyps by virtual chromoendoscopy is recognized as a key element for innovative imaging techniques. As a matter of facts diminutive colorectal polyps are very frequent and, if located in the rectosigmoid colon, they present a very low malignant risk (0.3% of evolution towards advanced adenoma and up to 0.08% of evolution towards invasive carcinoma). The real-time characterization would allow to identify the lowest risk polyps (hyperplastic subtype), to leave them *in situ* or, if resected, not to send them for histological examination, allowing a huge saving in healthcare associated costs.<br>Recently, the American Society for Gastrointestinal Endoscopy (ASGE) Technology Committee established the Preservation and Incorporation of Valuable endoscopic Innovations (PIVI) document, specific for real-time histological assessment for tiny colorectal polyps, to establish reference quality thresholds. Two performance standards have been developed to guide the use of advanced imaging:<br><br>1. for diminutive polyps to be resected and discarded without pathologic assessment, endoscopic technology (when used with high confidence) used to determine histology of polyps ≤ 5mm in size, when combined with the histopathology assessment of polyps > 5 mm in size, should provide a ≥ 90% agreement in assignment of post-polypectomy surveillance intervals when compared to decisions based on pathology assessment of all identified polyps;<br><br>2. in order for a technology to be used to guide the decision to leave suspected rectosigmoid hyperplastic polyps ≤ 5 mm in size in place (without resection), the technology should provide ≥ 90% negative predictive value (when used with high confidence) for adenomatous histology.<br><br>Computer-Aided-Diagnosis (CAD) is an artificial intelligence-based tool that would allow rapid and objective characterization of these lesions. The GI Genius CADx was developed to help endoscopists in their clinical practices for polyps characterization.
**6. Objective:**<br><br>Primary object of the study is to prospectively evaluate if GI Genius CADx output can achieve ≥ 90% Negative Predictive Value in characterization (i.e. as adenomas or non-adenomas) of diminutive rectosigmoid polyps (i.e. PIVI 2), having conventional histopathology analysis of the resected specimens as reference standard. An exploratory objective of the study is to assess if there is an agreement between the post-polypectomy surveillance intervals assigned by a combined use of GI Genius CADx as a means for optical diagnosis of diminutive (≤5 mm) rectosigmoid polyps and conventional histology of polyps >5 mm, and and those assigned by histology as unique means of diagnosis of all polyps (regardless of size or location).
**7. Study sites:**<br><br>Ospedale Nuovo Regina Margherita, Rome, Italy

Ospedale dei Castelli, Rome, Italy

8. Subject population:

Patients aged 40-80 undergoing colonoscopy

9. Inclusion/Exclusion criteria:

Inclusion criteria:

- Patients aged 40 years or older undergoing screening colonoscopy for CRC
- Ability to provide written, informed consent (approved by EC) and understand the responsibilities of trial participation.

Exclusion criteria:

- subject at high risk for CRC;
- subjects with a personal history of CRC, IBD or hereditary polyposis or non-polyposis syndromes;
- patients with previous resection of the sigmoid rectum;
- patients on anticoagulant therapy, which precludes resection / removal operations due to histopathological findings;
- patients who perform an emergency colonoscopy.

10. Study groups: Single-arm

11. Study procedure:

Each patient will undergo standard white-light colonoscopy with the support of the latest version of CE marked GI Genius available.

Each polyp identified during the colonoscopy procedure will be first prepared for being characterized as follows:

- white-light examination (use of virtual chromoendoscopy techniques [e.g. NBI, BLI, LCI] will not be allowed in this first step)
- no zoom
- washed properly
- framed at 6 o'clock
- framed at the nearest distance while keeping it on focus
- framed steadily

The endoscopist will move around the target polyp until GI Genius CADx will provide an optical diagnosis.

The endoscopist will be asked to predict polyp's histology at this stage and will categorize it as either adenoma or non-adenoma along with the confidence of his assessment. This first prediction will be recorded in the study CRF.

Furthermore, the GI Genius CADx optical diagnosis will be recorded on the study CRF.

Subsequently, the endoscopist will switch on blue light virtual chromoendoscopy (i.e. NBI or BLI) and will be asked to record in the CRF his endoscopic assessment of the polyp: again, the endoscopist will predict polyp's histology at this stage and will categorize it as either adenoma or non-adenoma along with the confidence of his assessment. This second prediction will be recorded in the study CRF. Additionally, endoscopist will record polyp morphology (according to Paris classification), size and location (Cecum, Ascending colon, Transverse colon, Descending colon, Sigmoid colon and Rectum).

All the procedure will be video recorded from the beginning to the end with a recorder directly attached to the endoscopy video processor (prior to GI Genius), so to record the video without any additional overlay (i.e. without GI Genius CADe overlay).

All polyps will be removed, collected separately each from the other and stored in formaline at 10% to be analyzed by the local pathologists. All lesions will be classified by the pathologist according to the classification of Vienna. An advanced adenoma is defined as adenoma of 10 mm and/or with a villous component > 20% and/or high-grade dysplasia. Histology results will be recorded in the study CRF.

After the procedure has been carried out and data provided by the endoscopist has been properly recorded, before the availability of the histology results, without the presence of the patient and the endoscopist, the video recording will be reviewed by a team of endoscopists to get their optical biopsies and confidence about polyp adenomatous or non-adenomatous histology.

12. Follow-up schedule:

There will be no patient follow-up

13. Timeline:

First patient: May 2021
End of Study: August 2021

14. Study endpoints:

Primary endpoint:

- Negative Predictive Value (NPV) of GI Genius CADx optical diagnosis on diminutive (≤5 mm) rectosigmoid polyps.

Exploratory endpoints:

1. Agreement in assignment of post-polypectomy surveillance intervals according to PIVI guidelines between:
    - the assignment identified according to the combined GI Genius CADx optical diagnosis for diminutive (≤5 mm) polyps and histology for larger polyps (> 5 mm), and
    - the assignment identified according to histology alone regardless of lesion size.
2. Sensitivity, Specificity, Accuracy, PPV and NPV of GI Genius CADx optical diagnosis
3. Sensitivity, Specificity, Accuracy, PPV and NPV of the optical diagnosis of endoscopists assessing lesions in white-light with the help of GI Genius CADx
4. Sensitivity, Specificity, Accuracy, PPV and NPV of the optical diagnosis of endoscopists assessing lesions with virtual chromoendoscopy and without the help of GI Genius CADx

15. Analysis plan:

1. Test if the lower bound of the 95% CI of the GI Genius CADx optical diagnosis NPV for diminutive (≤ 5 mm) rectosigmoid polyps is greater or equal than 90%.

2. Test if agreement in assignment of post-polypectomy surveillance intervals of the enrolled patients according to PIVI guidelines between:

    a. the combined use of GI Genius CADx as optical diagnosis for diminutive (≤5 mm) polyps and conventional histology as diagnostic tool for polyps > 5 mm, and

    b. histology as diagnostic tool for all the resected polyps (regardless of size).

    has a lower bound of the 95% CI greater of equal than 90%.

3. Report descriptive analysis of Sensitivity, Specificity, Accuracy, PPV and NPV of GI Genius CADx optical diagnosis
4. Report descriptive analysis of Sensitivity, Specificity, Accuracy, PPV and NPV of the optical diagnosis of endoscopists assessing lesions in white-light with the help of GI Genius CADx
5. Report descriptive analysis of Sensitivity, Specificity, Accuracy, PPV and NPV of the optical diagnosis of endoscopists assessing lesions with virtual chromoendoscopy and without the help of GI Genius CADx

16. Sample size:

A previous pilot study involving GI Genius CADx on 40 patients, reported an optical diagnosis of "non-adenoma" on 22 out of 23 assessed recto-sigmoid hyperplastic diminutive polyps, and could not reach an optical diagnosis on 1 polyp. The sample size calculation is therefore conservatively assuming that GI Genius CADx has an NPV between 95.6% (worst case analysis) and 100% on diminutive recto-sigmoid polyps and will therefore consider 97.8% as the expected NPV performance.

Using a one-sided alpha level of 0.025, a total of 148 subjects is required to achieve 80% power to meet the primary endpoint of the study, which is increased by 5% to account for drop-out rate. The number of subject to be enrolled will therefore be at least 156 patients.

Table of Contents

1 INTRODUCTION **10**

1.1 Background 10

1.2 Software-aided colon polyp detection 10

1.3 Brief description of the investigational medical device 12

2. STUDY RATIONALE AND DESIGN **12**

2.1 Study objective 12

2.2 Study rationale 13

2.3 Risks and benefits of the investigational device and of the clinical study 13

2.3.1 Anticipated clinical benefits 13

2.3.2 Risks associated with participation in the clinical study 13

2.4 Timeline 13

2.5 Sample size 13

2.6 Study sites 13

2.7 Principal investigator 14

2.8 Subinvestigator 14

3 STUDY POPULATION **14**

3.1 Target population 14

3.2 Inclusion Criteria 14

3.3 Exclusion Criteria 14

7. STUDY VISITS AND PROCEDURES **14**

8 DATA COLLECTION AND MANAGEMENT **19**

8.1 Case report form 19

8.2 Data collection 19

8.3 Unique subject identifier 19

9 STATISTICAL ANALYSIS **19**

10 STUDY ENDPOINTS **19**

10.1 Primary endpoint: 19

10.2 Exploratory endpoints: 19

11 ETHICAL CONSIDERATIONS **20**

11.1 Ethics and Good Clinical Practice 20

11.2 Informed Consent 20

11.3 Confidentiality 20
11.4 Subject withdrawal 20
11.5 Patient Safety 21
11.6 Adverse Events-Definitions 21
11.6.1 Unanticipated adverse device effect 21
11.6.2 Serious Adverse Event (SAE) 21
11.6.3 Serious injury 22
11.6.4 Eliciting adverse effect information. 22
11.6.5 AEs monitoring window 22
11.6.6 AEs recording 22
11.7 Adverse Event Reporting - General 24
12 STUDY TERMINATION 24
13 INFORMATION SECURITY 24
13.1 Data integrity and safety 24
13.2 Documentation retention and archiving 25
14 ADMINISTRATIVE PROCEDURES 26
14.1 Protocol amendments 26
15 REFERENCES 26

1 INTRODUCTION

1.1 Background

Diminutive colorectal polyps (≤ 5 mm) represent most of the polyps detected during colonoscopy, especially in the rectum-sigmoid tract. The characterization of these polyps by virtual chromoendoscopy is recognized as a key element for innovative imaging techniques. As a matter of facts diminutive colorectal polyps are very frequent and, if located in the rectosigmoid colon, they present a very low malignant risk (0.3% of evolution towards advanced adenoma and up to 0.08% of evolution towards invasive carcinoma). The real-time characterization would allow to identify the lowest risk polyps (hyperplastic subtype), to leave them in situ or, if resected, not to send them for histological examination, allowing a huge saving in healthcare associated costs.

Recently, the American Society for Gastrointestinal Endoscopy (ASGE) Technology Committee established the Preservation and Incorporation of Valuable endoscopic Innovations (PIVI) document, specific for real-time histological assessment for tiny colorectal polyps, to establish reference quality thresholds. Two performance standards have been developed to guide the use of advanced imaging:

1. for diminutive polyps to be resected and discarded without pathologic assessment, endoscopic technology (when used with high confidence) used to determine histology of polyps ≤ 5mm in size, when combined with the histopathology assessment of polyps > 5 mm in size, should provide a ≥ 90% agreement in assignment of post-polypectomy surveillance intervals when compared to decisions based on pathology assessment of all identified polyps;
2. in order for a technology to be used to guide the decision to leave suspected rectosigmoid hyperplastic polyps ≤ 5 mm in size in place (without resection), the technology should provide ≥ 90% negative predictive value (when used with high confidence) for adenomatous histology.

Computer-Aided-Diagnosis (CAD) is an artificial intelligence-based tool that would allow rapid and objective characterization of these lesions. The GI Genius CADx was developed to help endoscopists in their clinical practices for polyps characterization.

1.2 Software-aided colon polyp detection

GI Genius (also referred to as CB-17-08) CADe System is an Intelligent Endoscopy Module designed to be an adjunct to video colonoscopy with the purpose of supporting the operating physician with artificial intelligence during Colorectal Cancer Screening and Surveillance procedures.

To make GI Genius CADe System working, the module is placed on a shelf of a standard endoscopy tower and connected to both the video processor and the main display, both using the well-established SDI or HD-SDI video standards. The large part of the currently marketed endoscopy solutions is natively compatible with GI Genius CADe System, the few others can be connected using suitable adaptors.

As a result, the endoscopy video stream is flowing through the module with no modification or delay, without changing the way the endoscopy procedure is carried out.

At the same time, in real time, the module augments the video stream by adding small green overlay markers surrounding areas of interest, such that they can be further inspected and assessed independently according to the established standard clinical practice. Overlaid markers are further notified with a convenient sound.

GI Genius CADe System gives full control to the operating physician, being able to enable or disable the overlay markers at any time with a single touch.

Figure 1. GI Genius system on video endoscopy trolley.

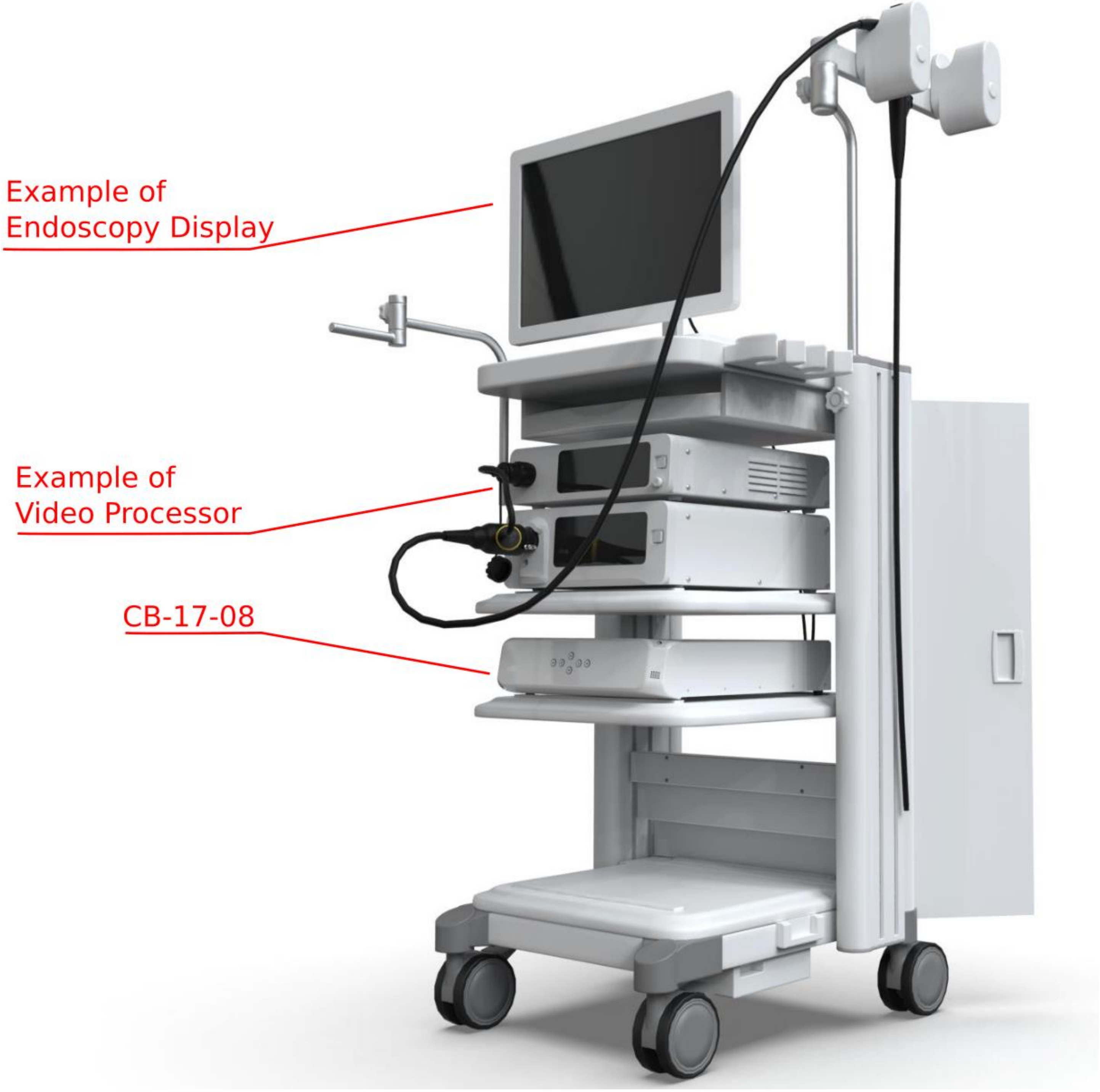

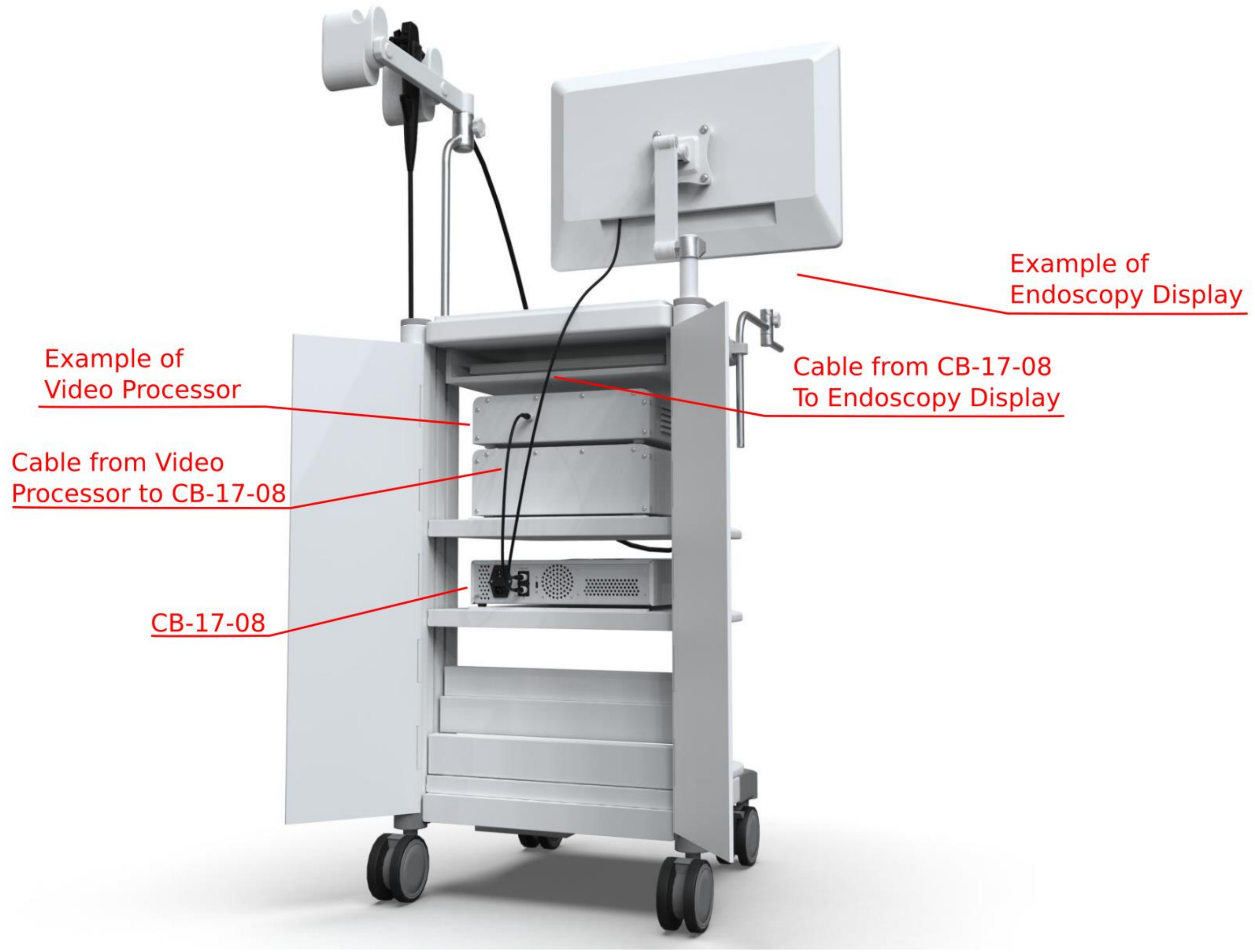

1.3 Brief description of the investigational medical device

GI Genius CADx System is a software that, when installed in GI Genius CADe System (in the same hardware module of the CADe system), will add the ability to provide a histology prediction of framed polyps during real-time colonoscopy, with each polyp being classified as adenoma or non-adenoma. If the device is not able to reach a prediction, a no-prediction message will be displayed.
GI Genius CADx System (version 3.0.0) has received the CE mark as a class IIa medical device by an European Notified Body under the Medical Device Directive 93/42/EEC and subsequent amendments. GI Genius CADx System can therefore be legally marketed in Europe, and the study will be considered post-marketing.

2. STUDY RATIONALE AND DESIGN

2.1 Study objective

Primary object of the study is to prospectively evaluate if GI Genius CADx output can achieve ≥ 90% Negative Predictive Value in characterization (i.e. as adenomas or non-adenomas) of diminutive rectosigmoid polyps (i.e. PIVI 2), having conventional histopathology analysis of the resected specimens as reference standard. An exploratory objective of the study is to assess if there is an agreement between the post-polypectomy

surveillance intervals assigned by a combined use of GI Genius CADx as a means for optical diagnosis of diminutive (≤5 mm) rectosigmoid polyps and conventional histology of polyps >5 mm, and and those assigned by histology as unique means of diagnosis of all polyps (regardless of size or location).

2.2 Study rationale

The real-time characterization by GI Genius CADx would allow to identify the lowest risk polyps (hyperplastic subtype), to leave them *in situ* or, if resected, not to send them for histological examination, allowing a huge saving in healthcare associated costs. The study will assess prospectively if the use of GI Genius CADx provides a useful tool to perform real-time characterizaiont of the colorectal polyps and if the device performances meet the PIVI guideline thresholds.

2.3 Risks and benefits of the investigational device and of the clinical study

2.3.1 Anticipated clinical benefits

The benefit for patients in participating in this study is that they will undergo with the most up-to-date GI Genius device, incorporating both detection (CADe) and characterization (CADx) features. There might also be a benefit for future patients, depending on the study results.

2.3.2 Risks associated with participation in the clinical study

No adjunctive risks are expected compared to standard endoscopic procedure. Endoscopy procedure will be performed according to the current best-practice.

2.4 Timeline

First patient is planned for enrollment on May 2021. The enrollment is expected to be completed in 4 months.

2.5 Sample size

A previous pilot study involving GI Genius CADx in 40 patients, reported an optical diagnosis of "non-adenoma" on 22 out of 23 assessed recto-sigmoid hyperplastic diminutive polyps, and could not reach an optical diagnosis on 1 polyp. The sample size calculation is therefore conservatively assuming that GI Genius CADx has an NPV between 95.6% (worst case analysis) and 100% on diminutive recto-sigmoid polyps and will therefore consider 97.8% as the expected NPV performance.

Using a one-sided alpha level of 0.025, a total of 148 subjects is required to achieve 80% power to meet the primary endpoint of the study. Anticipating a 5% drop-out rate, the target is to enroll at least 156 patients.

2.6 Study sites

Nuovo Regina Margherita Hospital, ASL Roma 1, Rome, Italy
Ospedale dei Castelli Hospital, ASL Roma 6, Ariccia, Rome, Italy

2.7 Principal investigator

Dott. Cesare Hassan (Nuovo Regina Margherita Hospital, Rome)

2.8 Subinvestigator

Dr. Giulio Antonelli (Ospedale dei Castelli Hospital, Ariccia, Rome).

3 STUDY POPULATION

3.1 Target population

Patients aged 40 years or older undergoing colonoscopy for screening of colorectal cancer (CRC).

3.2 Inclusion Criteria

- Patients aged 40 years or older undergoing screening colonoscopy for CRC;
- Ability to provide written, informed consent (approved by EC) and understand the responsibilities of trial participation.

3.3 Exclusion Criteria

- subjects undergoing screening colonoscopy as part of the regional screening program for CRC;
- subjects undergoing CRC surveillance colonoscopy;
- subject at high risk for CRC;
- subjects with a personal history of CRC, IBD or hereditary polyposic or non-polyposic syndromes;
- patients with previous resection of the sigmoid rectum;
- patients on anticoagulant therapy, which precludes resection / removal operations due to histopathological findings;
- patients who perform an emergency colonoscopy.

7. STUDY VISITS AND PROCEDURES

The following visits are foreseen for each subject:

1. **Screening Visit**

A screening visit is performed at the investigational site. During this visit out-patients scheduled for colonoscopy will be informed about the background of the proposed study, the aims, procedures, inclusion and exclusion criteria, benefits and possible risks of the study prior to signing the informed consent form for inclusion in the study. If participating to the study is of interest to the subject, the informed consent form (ICF) will be discussed and presented. The subject must sign the ICF prior to enrolment. Possible clinical or laboratory examinations will be done , according to the local standard procedures. No procedures beyond the standard procedures in preparation to colonoscopy are forseen per the current protocol. The date for the colonoscopy procedure to be performed at the investigational site will be scheduled. The screening visit can be performed on the same day of colonoscopy. The concomitant medications taken from the date of the informed consent up to the end of the colonoscopy visit will be recorded.

2. **Colonoscopy visit**

Each eligible patient meeting inclusion/exclusion criteria who have agreed to participate to the study and have signed the ICF will undergo a colonoscopy examination performed by an experienced endoscopist. All polyps (mucosal lesions) detected during the colonoscopy will be removed with standard techniques of polyp resection. Bowel preparation, including split dose, will be done according to the standard protocols of the individual sites. The quality of bowel preparation will be assessed during colonoscopy using the Boston Bowel Preparation Scale (BBPS). Sedation according to the sites best experience and standard procedures will be delivered to the patient. Prior to undergoing colonoscopy, study subjects will undergo placement of intravenous catheter. Sedative medications (Midazolam, Meperidine, Propofol, etc.) will be administered as per local institutional practice if applicable. Women of child bearing potential will undergo a poregnancy test to exclude pregnancy prior to the colonoscopy if foreseen by standard procedures at the investigational site.

Study procedure:

Each patient will undergo standard white-light colonoscopy with the support of the latest version of CE marked GI Genius available.

Each polyp identified during the colonoscopy procedure will be first prepared for being characterized as follows:

white-light examination (use of virtual chromoendoscopy techniques [e.g. NBI, BLI, LCI] will not be allowed in this first step)
no zoom
washed properly
framed at 6 o’clock
framed at the nearest distance while keeping it on focus
framed steadily

The endoscopist will move around the target polyp until GI Genius CADx will provide an optical diagnosis.

The endoscopist will be asked to predict polyp’s histology at this stage and will categorize it as either adenoma or non-adenoma along with the confidence of his assessment. This first prediction will be recorded in the study CRF.

Furthermore, the GI Genius CADx optical diagnosis will be recorded on the study CRF.

Subsequently, the endoscopist will switch on blue light virtual chromoendoscopy (i.e. NBI or BLI) and will be asked to record in the CRF his endoscopic assessment of the polyp: again, the endoscopist will predict polyp's histology at this stage and will categorize it as either adenoma or non-adenoma along with the confidence of his assessment. This second prediction will be recorded in the study CRF. Additionally, endoscopist will record polyp morphology (according to Paris classification), size and location (Cecum, Ascending colon, Transverse colon, Descending colon, Sigmoid colon and Rectum).

All the procedure will be video recorded from the beginning to the end with a recorder directly attached to the endoscopy video processor (prior to GI Genius), so to record the video without any additional overlay (i.e. without GI Genius CADe overlay).

All polyps will be removed, collected separately each from the other and stored in formaline at 10% to be analyzed by the local pathologists. All lesions will be classified by the pathologist according to the classification of Vienna. An advanced adenoma is defined as adenoma of 10 mm and/or with a villous component > 20% and/or high-grade dysplasia. Histology results will be recorded in the study CRF.

After the procedure has been carried out and data provided by the endoscopist has been properly recorded, before the availability of the histology results, without the presence of the patient and the endoscopist, the video recording will be reviewed by a team of endoscopists to get their optical biopsies and confidence about polyp adenomatous or non-adenomatous histology.

3. Endoscopic descriptions

For each polyp, the endoscopist will document if the polyp was characterized by the GI Genius CADx polyp characterization algorithm; additionally, the endoscopist will provide his own assessment of the predicted polyp histology, using the scale in Table 1 and Table 2.

Table 1. Endoscopist's assessment of the predicted polyp histology.

Hyperplastic polyp (HP)
Adenomatous polyp (AD)
Sessile Serrated Lesion (SSL)
Cancer (CA)
Other non-adenomatous polyps
Other/Undetermined (OU)

Table 2. Endoscopist's binary assessment of the predicted polyp histology.

Adenoma (ADE)
Non-adenoma (NOA)

The endoscopist will also record the level of confidence of his assessment, which will be categorized as High or Low.

In addition, the endoscopist will record the location, the endoscopic appearance (morphology) and size of each colon lesion (polyp). Polyp morphology will be described based on Paris endoscopic classification of colon polyps (see **Table 3** and **Figure 2** below).

The colon lesions (polyps) are classified into 3 groups. Protruded lesions are classified Type I lesions, flat and elevated lesions as type II lesions and excavated lesions as type III lesions. Lesions classified as type lesions will be considered polypoid lesions; lesions classified as either type II or type III lesions will be considered non-polypoid lesions.

Table 3. Paris classification categories.

**Category**	**Morphology**	**Description**
Polypoid	Ip	Protruded, Pedunculated
	Is	Protruded, Sessile
Non-polypoid	IIa	Superficial, elevated
	IIb	Flat
	IIc	Superficial shallow, depressed
	III	Excavated

Figure 2. Graphical representation of Paris classification categories.

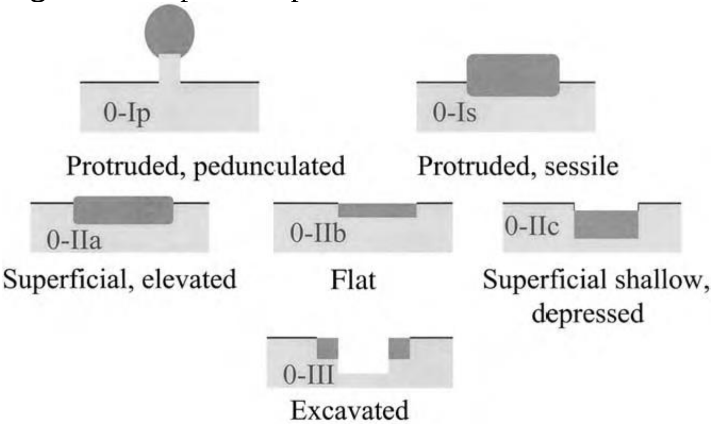

The size of each lesion will be estimated by the endoscopist and will be reported as diminutive (size 0-5 mm), small (size 6-9 mm) or large (size 10 mm or larger).
The validated Boston Bowel Preparation Score (BBPS) will be used to rate the colon cleansing and the presence of fluids in the colon [Lai, Calderwood, Doros, Fix, & Jacobson, 2009]. To apply the BBPS three broad regions of the colon are defined: the right colon (including the caecum and ascending colon), the transverse colon (including the hepatic and splenic flexures), and the left colon (including the descending colon, sigmoid colon, and rectum). Each of these three sections will be rated according to a 4-point scale (0-3) as follows:

0 unprepared colon segment with mucosa not seen due to solid stool that cannot be cleared
1 portion of mucosa of the colon segment seen, but other areas of the colon segment not well seen due to staining, residual stool and/or opaque liquid
2 minor amount of residual staining, small fragments of stool and/or opaque liquid, but mucosa of colon segment seen well
3 Entire mucosa of colon segment seen well with no residual staining, small fragments of stool or opaque liquid.

Each of the three regions of the colon defined above will receive a “segment score” from 0 to 3 and these segment scores will be summed up for a total BBPS score ranging from 0 to 9. Therefore, the maximum BBPS score for a perfectly clean colon without any residual liquid is 9 and the minimum BBPS score for an

unprepared colon is 0. If the Investigator aborts a procedure due to an inadequate preparation, then any non-visualized proximal segments are assigned a score of 0 [Lai, Calderwood, Doros, Fix, & Jacobson, 2009]. Aborted procedures due to inadequate preparation (the presence of fecal material in the cecum) will not be included in the primary endpoint analysis.

4. Histopathology assessment

The histopathological assessment of the resected or biopsied specimens collected during the study will be performed at the site's pathology laboratory.
The preparation of the slides of the biopsy specimens will be performed as per site's standard practice.
The pathologist will classify each resected or biopsied specimen according to the revised Vienna classification (**Table 4**), or to the serrated lesion classification (**Table 5**).

Table 4. The revised Vienna classification.

Category	Description
1	Negative for neoplasia
2	Indefinite for neoplasia
3	Mucosal low-grade neoplasia (low grade adenoma/dysplasia)
4	Mucosal high-grade neoplasia<br>4.1 High-grade adenoma/dysplasia<br>4.2 Non-invasive carcinoma (carcinoma *in situ*)[†]<br>4.3 Suspicious for invasive carcinoma<br>4.4 Intramucosal carcinoma[‡]
5	Submucosal invasion of neoplasia (carcinoma invading the submucosa or beyond)

[†]Non-invasive refers to the absence of evident invasion; [‡] Intramucosal refers to invasion into the lamina propria or muscularis mucosae.

Table 5. The serrated lesions classification

Category	Description
SSL[*]	Sessile serrated lesions[*]
TSA	Traditional serrated adenomas
HP	Hyperplastic polyps
FP	Fibroblastic polyps
MP	Mixed polyps (serrated part needs to be sub-defined into SSL, TSA, HP, FP)

[*] Sessile serrated lesion (SSL) is the updated denomination as per WHO 2019; these lesions were previously referred to as either Sessile Serrated Adenomas (SSA) or Sessile Serrated Polyps (SSP).

For conventional adenomas classified as either Vienna category 3 or 4.1, the histologist will also specify whether the adenomas are:

- Tubular adenomas
- Tubulovillous adenomas
- Villous adenomas

For Sessile serrated lesions, the histopathologist will also specify whether such lesions contain dysplasia or not.
If the analyzed specimes is not a colorectal polyp, the pathologist will specify which type of tissue it is (e.g. normal mucosa, inflammatory tissue, microlypoma, etc.).

8 DATA COLLECTION AND MANAGEMENT

8.1 Case report form

A case report form (CRF) will capture information regarding patient demographics, indication for procedure, recognition of endoscopic landmarks (caecum reached, colonoscopy is complete), procedure details and safety events, and all the data required for endpoint evaluation.

8.2 Data collection

The Investigator must ensure that the clinical data required by the study protocol are carefully reported in the CRFs within 72 h of visit completion. He must also check that the data reported in the CRFs correspond to those in the official files.

8.3 Unique subject identifier

The patient name will not be requested or registered by the Data Center. A sequential identified number will be automatically assigned to each patient registered in the study. Patient information or documentation should be considered "anonymous", and as such not subject to privacy laws, only when a "key" that allows patient identification is no longer available.

9 STATISTICAL ANALYSIS

Normally and non-normally distributed data will be presented using mean and standard deviation (SD) and median and interquartile ranges (IQR), respectively. Comparisons of means and proportions will be performed by Student's t test or chi-square test, as appropriate. For all comparisons, a p value of $\leq$ than 0.05 will be considered statistically significant.

10 STUDY ENDPOINTS

10.1 Primary endpoint:

- Negative Predictive Value (NPV) of GI Genius CADx optical diagnosis on diminutive (≤5 mm) rectosigmoid polyps.

10.2 Exploratory endpoints:

1. Agreement in assignment of post-polypectomy surveillance intervals of the enrolled patients according to established guidelines between:

   - the combined use of GI Genius CADx as optical diagnosis for diminutive (≤5 mm) polyps and conventional histology as diagnostic tool for polyps > 5 mm,

   and

   - histology as diagnostic tool for all the resected polyps (regardless of size or location).

2. Sensitivity, Specificity, Accuracy, PPV and NPV of GI Genius CADx optical diagnosis.
3. Sensitivity, Specificity, Accuracy, PPV and NPV of the optical diagnosis of endoscopists assessing lesions in white-light with the help of GI Genius CADx.
4. Sensitivity, Specificity, Accuracy, PPV and NPV of the optical diagnosis of endoscopists assessing lesions with virtual chromoendoscopy and without the help of GI Genius CADx.

11 ETHICAL CONSIDERATIONS

11.1 Ethics and Good Clinical Practice

The study coordinator ensures that this study is conducted in accordance with the Helsinki Declaration (amendments of Tokyo, Venice, Hong Kong, Somerset West, Edinburgh, Washington, Tokyo and Seoul) or with Italian laws and regulations.
The study will be conducted in accordance with the current good clinical practices (GCP).
The protocol and its annexes are subject to review and approval of the Independent Ethical Committee of competence.

11.2 Informed Consent

Each subject will have the nature and the purpose of this investigation explained to him or her by the Investigator or another member of the investigative team at the site. The study will be explained to the subjects in lay language. Prior to entry into this investigation the subject must give voluntary, written informed consent to participate by signing the consent form. On the same occasion, a member of the research team will also sign the informed consent form.
The original copy of the signed consent form will be kept in the Investigator file at the study site.

11.3 Confidentiality

All records identifying the subject must remain confidential and, with the limits permitted by applicable laws, are not made available to the public.

11.4 Subject withdrawal

The patient may refrain from participating in the study or withdraw consent to participate at any time. Any withdrawal will have no impact on the standard patient care standard. Patient participation may be interrupted at any time during the study without implications for further medical treatment if:

- He does not want to participate anymore
- The doctor believes that it is in their interest to stop participating in this study.
- The BBPS assessed by the Investigator is lower than 2 in any of the bowel colonic segments.

11.5 Patient Safety

Patients' vital signs, such as blood pressure, heart rate, and oxygen saturation, will be monitored during the endoscopy as per site's standard practice. Endoscopic procedures will be performed in the GI endoscopy laboratory.All necessary efforts to reduce the risk of discomfort or distress will be taken. Study staff will be particularly aware of the need to remain sensitive and respectful to participants. All patients will be provided with a direct telephone number to contact the Principal Investigator in the event of questions and symptoms. Careful data monitoring and quality control will be maintained with all possible precautions taken to protect the confidentiality of study subjects. All information with patient identifiers will be coded using study IDs and anonymized by deletion of all printed information. Patient information with identifiers will be under lock-and key, and access will be granted only to the PI and research co-coordinator.

11.6 Adverse Events-Definitions

Any untoward medical occurrence, unintended disease or injury, untoward clinical signs (including abnormal laboratory findings) in subjects, users or other persons, whether or not related to the medical device.

Notes:

This definition includes events related to the procedure involved.

11.6.1 Unanticipated adverse device effect

Means any serious adverse effect on health or safety or any life-threatening problem or death caused by, or associated with, a device, if that effect, problem, or death was not previously identified in nature, severity, or degree of incidence in the investigational plan or application (including a supplementary plan or application), or any other unanticipated serious problem associated with a device that relates to the rights, safety, or welfare of subjects.

11.6.2 Serious Adverse Event (SAE)

Adverse event that:

a) led to a death,
b) led to a serious deterioration in health that either:
    1) resulted in a life-threatening illness or injury, or
    2) resulted in a permanent impairment of a body structure or a body function, or
    3) required in-patient hospitalization or prolongation of existing hospitalization, or
    4) resulted in medical or surgical intervention to prevent life threatening illness orinjury or permanent impairment to a body structure or a body function.

c) led to fetal distress, fetal death or a congenital abnormality or birth defect.

NOTE 1: This includes device deficiencies that might have led to a serious adverse event if: (a) suitable action had not been taken or (b) intervention had not been made or (c) if circumstances had been less fortunate.

NOTE 2: A planned hospitalization for pre-existing condition, or a procedure required by the Clinical Investigation Plan, without a serious deterioration in health, is not considered to be a serious adverse event.

11.6.3 Serious injury

Means an injury or illness that:
(1) Is life-threatening,
(2) Results in permanent impairment of a body function or permanent damage to a body structure, or
(3) Necessitates medical or surgical intervention to preclude permanent impairment of a body function or permanent damage to a body structure.
Permanent means irreversible impairment or damage to a body structure or function, excluding trivial impairment or damage.
Caused or contributed means that a death or serious injury was or may have been attributed to a medical device, or that a medical device was or may have been a factor in a death or serious injury, including events occurring as a result of:
(1) Failure;
(2) Malfunction;
(3) Improper or inadequate design;
(4) Manufacture;
(5) Labelling; or
(6) User error.

11.6.4 Eliciting adverse effect information.

Clinical study subjects will be routinely questioned about adverse effects at study visits.

The clinical Investigator is responsible for reporting any observed events which meet the definition of adverse event into each patients CRF.

For any SAE or serious injury occurring in the study, the participating Investigators (Principal and subinvestigators) will have to document the event in a written narrative, and to establish a potential relationship between the event itself and the use of study device.

11.6.5 AEs monitoring window

- Start of monitoring: from immediately after the signature of the informed consent
- End of monitoring: last follow-up visit/ETV

An AE occurring after the last follow-up visit/ETV and coming to knowledge of the Investigator (e.g. by spontaneous reporting by study subjects) must be recorded only if it is an AR, according to the Investigator’s judgment.

11.6.6 AEs recording

All AEs derived by spontaneous, unsolicited reports of the subjects, by observation and by routine open questioning should be collected and reported in each subjects CRF.

The following minimal information will be recorded for an AE (detailed explanation for each element is available in the SOP or in the operative summary made available to the clinical center) in the source documents and later transcribed into the CRF:

1. Adverse Event: progressive number of the adverse event

2. Description: verbatim description of the adverse event or
Follow-up: progressive number of follow-up of the adverse event

3. Start Date/Time: start date/time of the adverse event or
Follow-up Date/Time: follow-up date/time of the adverse event

4. End Date/Time: end date/time of the adverse event

5. Affected Body Area: anatomical location relevant for the event

6. Whether the adverse event start before or after the first use of the device or whether the adverse event has worsened or not after the first use of the study device.

7. Device Administration Date/Time Before Onset: if the adverse event started after the first use of the study device, the date/time of last use of the study device before the onset of the adverse event or Last Study device Administration Date/Time Before Worsening: In case of treatment emergent adverse event, the date/time of the last administration of the study device before the worsening of the adverse event.

8. Investigator's opinion about the reasonable possibility of a causal relationship with the study device.

9. Investigator's opinion about other causal relationship to the administration of the device

10. Severity: the severity or intensity of the event

- 1 Mild
- 2 Moderate
- 3 Severe

11. Pattern: Used to indicate the pattern of the event over time

- 1 Single Event
- 2 Continuous
- 3 Intermittent

12. Concomitant Therapy: if a concomitant therapy is given, it must be reported in the specific CRF forms

13. Study Discontinuation: if the adverse event causes the subject to be discontinued from the study

14. Other Action Taken: other actions taken because of the event

15. Outcome: Outcome of the event

- 1 Recovered/Resolved
- 2 Recovered/Resolved With Sequelae
- 3 Recovering/Resolving
- 4 Not Recovered/Not Resolved
- 5 Fatal
- 6 Unknown

11.7 Adverse Event Reporting - General

The primary Investigator will monitor any adverse events and device deficiency reported to the study staff and make an assessment, and determine if it's related to the study procedures including standard colonoscopy. All adverse events will be monitored until a satisfactory resolution and will be reported to the EC within 5 days of knowledge.
Within 3 days from occurrence, the Investigator must report to the manufacturer of the device any event, per Investigator's assessment, reasonably suggest that the study device has or may have caused an SAE or contributed to a death or serious injury. The Investigator shall notify the competent Ethics Committee (EC) of any serious injury or SAE with lethal outcome which occurred during a study. If the Investigator is initially unable to obtain all the necessary details for completing the narrative, he/she should in any case transmit all the available information. The Investigator should provide an appropriate follow-up of SAEs to all concerned parties.

Seriousness and causality must be assessed by the Investigator and recorded in the patients CRF.

12 STUDY TERMINATION

Any subject who wishes to withdraw from this investigation on his/her own accord and for whatever reason is entitled to do so without obligation and prejudice to further treatment. In addition, the Investigator may decide for reasons of medical prudence, to withdraw a subject. In either event, the Investigator will clearly document the date and reason(s) for the subject's withdrawal from this investigation in the CRF and should indicate whether he considers it was related to the study interventions.

13 INFORMATION SECURITY

13.1 Data integrity and safety

All paper charts pertaining to the patient will be kept under lock and key in coordinators office away from the endoscopy area. Only approved personnel by the EC will have access to the file storage.

To protect subject confidentiality, each subject will be assigned a case study number. The subject's name is not to appear anywhere on the Case Report Forms (CRF's) or supporting documentation. A study log with the identifiable information will be kept in a separate folder to enable the Investigators to assist in any research audit. No procedural data except the date of examination will be entered in to this log. All anonymized digital images recorded during the study will be retained in a secure access-controlled database.

13.2 Documentation retention and archiving

The Investigator-Sponsor will maintain records in accordance with Good Clinical Practice guidelines; to include:

- EC correspondence (including approval notifications) related to the clinical protocol; including copies of adverse event reports and annual or interim reports
- Current and past versions of the EC-approved clinical protocol and corresponding EC-approved consent form(s) and, if applicable, subject recruitment advertisements.
- Signed Investigator's Agreements;
- Curriculum vitae of the Investigators;
- Instructions for on-site preparation and handling of the device and/or study treatment or diagnostic product(s), and other study-related materials (i.e., if not addressed in the clinical protocol);
- Signed informed consent forms for each patient.
- Completed Case Report Forms;
- Source Documents or certified copies of Source Documents;
- Copies of Investigator-Sponsor correspondence to sub-Investigators, including notifications of adverse effect information;
- Subject screening and enrolment logs;
- Subject identification code list;
- Study delegation log, with indication of the study staff involved in the research activities and their role and responsibilities in the study.

The Investigator must keep source documents for each subject in the study. All information on the CRFs must be traceable to these source documents, which are generally stored in the subject's medical file. The source documents should contain all demographic and medical information, including laboratory data, etc., and the original signed informed consent forms.
Data reported in the CRF that are derived from source documents should be consistent with the source documents or the discrepancies should be explained.
The Investigator should maintain the study documents as specified in the relevant regulations and guidelines. These are documents which individually and collectively permit evaluation of a study and the quality of the data produced and include groups of documents, generated before the study commences, during the clinical study, and after termination of the study and include, but are not limited to, study protocol, amendments, submission and approval of EC, raw data of subjects including lab tests, insurance contracts, signed informed consent forms, e-CRFs, curricula vitae of the Investigators and other participants in the study, study staff lists and responsibilities, monitoring reports and final study report.
The Investigator should take measures to prevent accidental or premature destruction of these documents.
Study documents must be retained by the Investigator as long as needed to comply with national and international regulations.

14 ADMINISTRATIVE PROCEDURES

14.1 Protocol amendments

To obtain interpretable results, the Investigator will not alter the study conditions agreed upon and set out in this protocol. Amendments should be submitted prior to applying any modification to the study design and procedures. Any amendment must be set out in writing, giving the reasons, and being signed by all concerned parties. The amendment becomes then part of the protocol.

All amendments will be sent to the EC for approval, which will establish whether to submit them to the next EC review prior to implementation (e.g. for changes increasing the risk for subjects) or to consider them as simple notifications (e.g. for minor protocol changes or when the changes involve only logistical or administrative aspects of the trial).

15 REFERENCES

1. Rondonotti E, Paggi S, Amato A, et al. Blue-light imaging compared with high-definition white light for real-time histology prediction of colorectal polyps less than 1 centimeter: a prospective randomized study. Gastrointest Endosc. 2019 Mar;89:554-564.
2. Picot J, Rose M, Cooper K, et al. Virtual chromoendoscopy for the real-time assessment of colorectal polyps in vivo: a systematic review and economic evaluation. Health Technol Assess. 2017;21:1-308.
3. Ponugoti PL, Cummings OW, Rex DK. Risk of cancer in small and diminutive colorectal polyps. Dig Liver Dis. 2017;49:34-37.
4. Rex DK, Kahi C, O’Brien M et al. The American Society for Gastrointestinal Endoscopy PIVI (Preservation and Incorporation of Valuable Endoscopic Innovations) on real-time endoscopic assessment of the histology of diminutive colorectal polyps. Gastrointest Endosc 2011;73: 419–422.
5. Lieberman DA, Rex DK, Winawer SJ et al. Guidelines for colonoscopy surveillance after screening and polypectomy: a consensus update by the US Multi-Society Task Force on Colorectal Cancer. Gastroenterology 2012;143:844-857.
6. Hassan C, Quintero E, Dumonceau JM et al. Post-polypectomy colonoscopy surveillance: European Society of Gastrointestinal Endoscopy (ESGE) Guideline. Endoscopy 2013;45:842-51.
7. Neumann H, Neumann Sen H, Vieth M, et al. Leaving colorectal polyps in place can be achieved with high accuracy using blue light imaging (BLI). United European Gastroenterol J. 2018;6:1099-1105.
8. Rondonotti E, Hassan C, Andrealli A, et al. Clinical Validation of BASIC Classification for the Resect and Discard Strategy for Diminutive Colorectal Polyps. Clin Gastroenterol Hepatol. 2020 [Epubahead of print].
9. Mori Y, Kudo SE, Misawa M, et al, Simultaneous detection and characterization of diminutive polyps with the use of artificial intelligence during colonoscopy. VideoGIE. 2019 Jan 1;4(1):7-10. doi: 10.1016/j.vgie.2018.10.006. eCollection 2019 Jan.
10. Mori Y, Kudo SE, East JE, et al Cost savings in colonoscopy with artificial intelligence-aided polyp diagnosis: an add-on analysis of a clinical trial (with video).Gastrointest Endosc. 2020 Mar 30:S0016-5107(20)34034-7. doi: 10.1016/j.gie.2020.03.3759. Online ahead of print.

Roma, 06 Maggio 2021
Prot. n. 611/CE Lazio 1

Dott. Cesare Hassan
cesareh@hotmail.com

Oggetto: parere favorevole sullo studio interventistico di cui al Protocollo CHANGE nella versione 1.5_08/04/2021.

Il Comitato Etico Lazio 1, istituito con deliberazione n. 146 del 12 giugno 2013 della Regione Lazio e costituito con delibera n. 880 del 19 luglio 2013 dall'Azienda Ospedaliera San Camillo–Forlanini, ai sensi del Decreto Legge 13 Settembre 2012 n. 158 e in ottemperanza ai requisiti minimi di cui al Decreto 8 Febbraio 2013 del Ministero della Salute, rinnovato con delibera 968 del 04/08/2016, prorogato con Determinazione Regione Lazio n. G07729 del 06 Giugno 2019 fino al 31 Dicembre 2019, successivamente prorogato con deliberazione n. 0275 dell'A.O. San Camillo Forlanini del 25/02/2020 in attuazione della Determinazione Regione Lazio n. G17833 del 17 Dicembre 2019, si è riunito il giorno 28 Aprile 2021 alle ore 15,00 tramite piattaforma Go to meeting per discutere l'ordine del giorno, trasmesso per via telematica il giorno 22/04/2021 prot. n. 529/CE Lazio 1, con allegati i documenti inerenti alle sperimentazioni cliniche e gli emendamenti proposti:

- Comunicazioni del Presidente;
- Uso terapeutico;
- Audizione degli sperimentatori per gli studi di seguito elencati:
  - sperimentazioni cliniche;
  - studi osservazionali;
  - emendamenti ai protocolli di studio;
- Varie ed eventuali.

Sono presenti i seguenti componenti del CE Lazio 1: Prof.ssa Paola Grammatico, Dott.ssa Teresa Calamia, Dr. Mauro Calvani, Dr.ssa Anna Ceccorulli, Dr. Alberto Chiriatti, Dr.ssa Serena Fattori,Dr.ssa Diana Giannarelli, Prof. Francisco Javier Fiz Perez, Dr. Francesco Meo, Dr. Marco Montanaro, Prof. Paola Patrignani, Prof. Cosimo Prantera, Dr.ssa Susanna Ricci, Dr.ssa Domenica Tassielli, Dr. Marco Tubaro, Dr. Giovanni Maria Vincentelli.

Sono presenti i seguenti componenti della Segreteria Tecnico-Scientifica: Dr.ssa Giorgia Bandiera, Dr Pier Vittorio Lorizio.

Il Presidente, prof.ssa Paola Grammatico, alle ore 15.00 apre la web conference e verificato il numero legale (16 su 16) inizia la seduta.

...omissis...

Il Comitato Etico vista l'istanza del dott. Cesare Hassan dell'UO Medicina Materno-Fetale dell'Ospedale Cristo RE, del 08/04/2021 pervenuta il 26/04/2021 Prot. n. 550/CE Lazio 1, avente in oggetto la richiesta per l'autorizzazione alla conduzione dello studio interventistico con dispositivo medico post market secondo il Protocollo dal titolo "CHANGE – Characterization helping the assessment of colorectal neoplasia in gastrointestinal endoscopy", versione 1.5_08/04/2021, nell'UOC Gastroenterologia del PO Nuovo Regina Margherita – ASL Roma 1, sotto la sua responsabilità in qualità di sperimentatore principale nonché di promotore, con in allegato i seguenti documenti:

- Lettera di intenti del 08/04/2021

- Clinical Study Protocol dal titolo "CHANGE – Characterization helping the assessment of colorectal neoplasia in gastrointestinal endoscopy" nella versione 1.5_08/04/2021
- Sinossi dello studio in italiano
- Declaration of conformity: certificato EPT 0477.MDD.21/4179.1 / dispositivo medico GI GENIUS medical software – CB-17-08 software
- Product Specifications CONFIDENTIAL LG19110901 Rev. No. 3.0 Oct 01, 2020
- Appendix – The Histological Examination of Colorectal Polyps in Trial Change
- Documento sulle informazioni per il paziente e sul consenso - Consenso Informato v 2.0 del 08/04/2021
- Domanda dello sperimentatore per l'autorizzazione
- CV C. Hassan
- Dichiarazione pubblica sul conflitto di interesse dello sperimentatore

Il Comitato etico, al termine della discussione, verificata la documentazione allegata all'istanza del Richiedente del 08/04/2021, pervenuta il 26/04/2021 Prot. n. 550/CE Lazio 1, avente in oggetto la richiesta per l'autorizzazione alla conduzione dello studio interventistico con dispositivo medico post market secondo il Protocollo dal titolo "CHANGE – Characterization helping the assessment of colorectal neoplasia in gastrointestinal endoscopy", versione 1.5_08/04/2021, nell'UOC Gastroenterologia del PO Nuovo Regina Margherita – ASL Roma 1, sotto la sua responsabilità in qualità di sperimentatore principale e di promotore. Contestualmente chiede che sia inserito un numero telefonico attivo almeno 12 ore al giorno, oltre quelli di istituto già presenti, per garantire la reperibilità nel caso si verificassero le complicanze di cui al paragrafo "Rischi" del modulo di Consenso Informato v 2.0 del 08/04/2021.

Il Presidente del Comitato Etico Lazio 1
Prof.ssa Paola Grammatico
