# Supplementary material for: A novel AI device for real-time optical characterization of colorectal polyps
Source: NPJ Digit Med. 2022 Jun 30;5:84. doi: 10.1038/s41746-022-00633-6 (PMC9247164; doi:10.1038/s41746-022-00633-6)
Supplement: Supplementary file 3 — Reporting Summary Checklist [file 41746_2022_633_MOESM3_ESM.pdf]

nature portfolio

Corresponding author(s): Andrea Cherubini

Last updated by author(s): May 2, 2022

Reporting Summary

Nature Portfolio wishes to improve the reproducibility of the work that we publish. This form provides structure for consistency and transparency in reporting. For further information on Nature Portfolio policies, see our Editorial Policies and the Editorial Policy Checklist.

Statistics

For all statistical analyses, confirm that the following items are present in the figure legend, table legend, main text, or Methods section.

n/a	Confirmed	
☐	☒	The exact sample size (*n*) for each experimental group/condition, given as a discrete number and unit of measurement
☐	☒	A statement on whether measurements were taken from distinct samples or whether the same sample was measured repeatedly
☐	☒	The statistical test(s) used AND whether they are one- or two-sided<br>*Only common tests should be described solely by name; describe more complex techniques in the Methods section.*
☒	☐	A description of all covariates tested
☐	☒	A description of any assumptions or corrections, such as tests of normality and adjustment for multiple comparisons
☐	☒	A full description of the statistical parameters including central tendency (e.g. means) or other basic estimates (e.g. regression coefficient) AND variation (e.g. standard deviation) or associated estimates of uncertainty (e.g. confidence intervals)
☐	☒	For null hypothesis testing, the test statistic (e.g. F, t, r) with confidence intervals, effect sizes, degrees of freedom and P value noted<br>*Give P values as exact values whenever suitable.*
☒	☐	For Bayesian analysis, information on the choice of priors and Markov chain Monte Carlo settings
☒	☐	For hierarchical and complex designs, identification of the appropriate level for tests and full reporting of outcomes
☒	☐	Estimates of effect sizes (e.g. Cohen's *d*, Pearson's *r*), indicating how they were calculated

Our web collection on statistics for biologists contains articles on many of the points above.

Software and code

Policy information about availability of computer code

Data collection	OpenClinica for collecting eCRF (OC4 Stack 12.2) ; Atomos Shogun Inferno for recording endoscopy videos
Data analysis	R for statistical analysis (version 4.1.2)

For manuscripts utilizing custom algorithms or software that are central to the research but not yet described in published literature, software must be made available to editors and reviewers. We strongly encourage code deposition in a community repository (e.g. GitHub). See the Nature Portfolio guidelines for submitting code & software for further information.

Data

Policy information about availability of data

All manuscripts must include a data availability statement. This statement should provide the following information, where applicable:

- Accession codes, unique identifiers, or web links for publicly available datasets
- A description of any restrictions on data availability
- For clinical datasets or third party data, please ensure that the statement adheres to our policy

De-identified study data may be made available at publication upon request to the corresponding author. Data sharing will only be available for academic research, instead of commercial use or other objectives. A data use agreement and institutional review board approval will be required as appropriate.

Field-specific reporting

Please select the one below that is the best fit for your research. If you are not sure, read the appropriate sections before making your selection.

☒ Life sciences ☐ Behavioural & social sciences ☐ Ecological, evolutionary & environmental sciences

For a reference copy of the document with all sections, see nature.com/documents/nr-reporting-summary-flat.pdf

Life sciences study design

All studies must disclose on these points even when the disclosure is negative.

Sample size	A previous pilot study involving GI Genius CADx on 60 patients reported an accuracy of 85%. The sample size for Standalone CADx study was calculated assuming that experts can perform optical characterization with an accuracy of 87%. Using a one-sided alpha level of 0.025, a total of 480 lesions is required to achieve 80% power, which is increased by 5% to account for dropouts. The minimum number of polyps needed for Standalone CADx study was therefore determined to be 504. Since CHANGE study collected a total of 513 polyps with a valid video recording and a valid histopathology outcome, all these polyps were included in the Standalone CADx statistical analysis. (Paragraph 4.3.4)
Data exclusions	Polyps for which video recording failed or for which no histology could be obtained were excluded. (Paragraph 4.2.1)
Replication	The manuscript describes the result of a multi-reader study performed on prospectively acquired data.
Randomization	To reach the target number of reviewers, 20 invitations were sent considering a 20% dropout. However, 10 additional invitations were needed and a final number of 10 experts and 11 non-experts reviewers was reached. Videos were shown in a randomized order to each endoscopists via a dedicated secure website. (Paragraph 4.2.2)
Blinding	Endoscopists were blinded to histology and CADx results and a green box was manually drawn (overlaid) around the target polyp in each videoclip frame to remove any ambiguity in the identification of the region of interest. (Paragraph 4.2.2)

Reporting for specific materials, systems and methods

We require information from authors about some types of materials, experimental systems and methods used in many studies. Here, indicate whether each material, system or method listed is relevant to your study. If you are not sure if a list item applies to your research, read the appropriate section before selecting a response.

Materials & experimental systems

n/a	Involved in the study	
☒	☐	Antibodies
☒	☐	Eukaryotic cell lines
☒	☐	Palaeontology and archaeology
☒	☐	Animals and other organisms
☒	☐	Human research participants
☐	☒	Clinical data
☒	☐	Dual use research of concern

Methods

n/a	Involved in the study	
☒	☐	ChIP-seq
☒	☐	Flow cytometry
☒	☐	MRI-based neuroimaging

Clinical data

Policy information about clinical studies

All manuscripts should comply with the ICMJE guidelines for publication of clinical research and a completed CONSORT checklist must be included with all submissions.

Clinical trial registration	NCT04884581
Study protocol	https://osf.io/m5cxt
Data collection	The CHANGE study (“Characterization Helping in the Assessment of Neoplasia in Gastrointestinal Endoscopy”, ClinicalTrials.gov NCT04884581), a single-center, single-arm, prospective study acquired high resolution videos of colonoscopy procedures conducted using GI Genius CADx v3.0 from May 2021 until July 2021. The study was approved by the local Institutional Review Board (Comitato Etico Lazio 1, prot. 611/CE Lazio1) and conducted in accordance with the Declaration of Helsinki. Before participation, all participants provided written informed consent. The 165 patients screened in the CHANGE study were considered for the Standalone CADx study (“Standalone Performances of Artificial Intelligence CADx for Optical Characterization of Colorectal Polyps”, https://osf.io/m5cxt), a study aiming at assessing the standalone performance of the CADx and whose results are reported in this manuscript. All the colonoscopy videos considered in the study were acquired in full length with unaltered quality, bearing no trace of the AI used (no overlay). Patients clinical data and polyp histopathological information were saved in an electronic Case Report Form (eCRF). The localization of each polyp in each patient was carefully annotated by scientific annotation experts. This was confronted with data in the eCRF for the same patient to avoid any possibility of erroneous correspondence between polyp in the video and the related

histology. Polyps for which video recording failed or for which no histology could be obtained were excluded. For each polyp, a short video clip was prepared, starting a few seconds before the first polyp appearance and ending with polyp endoscopic resection. If multiple polyps were present in the same video section, a separate clip was generated for each individual polyp. This resulted in a total of 513 videoclips, 198 adenomas and 315 non-adenomas.

Outcomes

The analysis for the primary endpoint was to assess if the lower bound of 95% CI for difference in accuracies (CADx_WL - Experts) is higher than -10%. The analysis for the first exploratory endpoint was to assess if the lower bound of 95% CI for difference in accuracies (CADx_WL - Non-Experts) is greater than 0. The analysis for the second exploratory endpoint was to assess if the lower bound of 95% CI for difference in accuracies (CADx_BL - CADx_WL) is greater than -10%.
